# The association between the stress hyperglycaemia ratio and mortality in cardiovascular disease: a meta-analysis and systematic review

**DOI:** 10.1186/s12933-024-02454-1

**Published:** 2024-11-16

**Authors:** Harriet Esdaile, Shaila Khan, Jamil Mayet, Nick Oliver, Monika Reddy, Anoop S. V. Shah

**Affiliations:** 1 Faculty of Medicine, Department of Metabolism, Digestion and Reproduction, Imperial Centre for Translational and Experimental Medicine, Imperial College London, London, UK; 2 Faculty of Medicine, National Heart and Lung Institute, Imperial College London, London, UK; 3 Department of Cardiology, Imperial College Healthcare NHS Trust, London, UK; 4 Department of Non Communicable Disease Epidemiology, London School of Hygiene and Tropical Medicine, London, UK

**Keywords:** Hyperglycaemia, Cardiodiabetology, Stress hormones, Mortality, Inpatient Management

## Abstract

**Background:**

A raised stress hyperglycaemia ratio (SHR) has been associated with all-cause mortality and may better discriminate than an absolute glucose value. The aim of this meta analysis and systematic review is to synthesise the evidence assessing the relationship between the SHR and all-cause mortality across three common cardiovascular presentations.

**Methods:**

We undertook a comprehensive search of Medline, Embase, Cochrane CENTRAL and Web of Science from the date of inception to 1st March 2024, and selected articles meeting the following criteria: studies of patients hospitalised for acute myocardial infarction, ischaemic stroke or acute heart failure reporting the risk (odds ratio or hazard ratio) for all-cause mortality associated with the SHR. A random effects model was used for primary analysis. Subgroup analysis by diabetes status and of mortality in the short and long term was undertaken. Risk of bias assessment was performed using the Newcastle Ottawa quality assessment scale.

**Results:**

A total of 32 studies were included: 26 studies provided 31 estimates for the meta-analysis. The total study population in the meta analysis was 80,010. Six further studies were included in the systematic review. Participants admitted to hospital with cardiovascular disease and an SHR in the highest category had a significantly higher risk ratio of all-cause mortality in both the short and longer term compared with those with a lower SHR (RR = 1.67 [95% CI 1.46–1.91], p < 0.001). This finding was driven by studies in the myocardial infarction (RR = 1.75 [95% CI 1.52–2.01]), and ischaemic stroke cohorts (RR = 1.78 [95% CI 1.26–2.50]). The relationship was present amongst those with and without diabetes (diabetes: RR 1.49 [95% CI 1.14–1.94], p < 0.001, no diabetes: RR 1.85 [95% CI 1.49–2.30], p < 0.001) with p = 0.21 for subgroup differences, and amongst studies that reported mortality as a single outcome (RR of 1.51 ([95% CI 1.29–1.77]; p < 0.001) and those that reported mortality as part of a composite outcome (RR 2.02 [95% CI 1.58–2.59]; p < 0.001). On subgroup analysis by length of follow up, higher SHR values were associated with increased risk of mortality at 90 day, 1 year and > 1year follow up, with risk ratios of 1.84 ([95% CI 1.32–2.56], p < 0.001), 1.69 ([95% CI 1.32–2.16], p < 0.001) and 1.58 ([95% CI 1.34–1.86], p < 0.001) respectively.

**Conclusions:**

A raised SHR is associated with an increased risk of all-cause mortality following myocardial infarction and ischaemic stroke. Further work is required to define reference values for the SHR, and to investigate the potential effects of relative hypoglycaemia. Interventional trials targeting to the SHR rather than the absolute glucose value should be undertaken.

**PROSPERO database registration:**

CRD 42023456421 https://www.crd.york.ac.uk/prospero/display_record.php?ID=CRD42023456421

**Supplementary Information:**

The online version contains supplementary material available at 10.1186/s12933-024-02454-1.

## Introduction

Hyperglycaemia, alongside other factors, is associated with worse short-term and long-term outcomes in patients admitted with acute myocardial infarction (AMI) [1] and acute ischaemic stroke (AIS) [2]. Contributors include the stress hyperglycaemia phenomenon, treatment (including the omission of diabetes related medications because of vomiting or peri-intervention, and use of supplemental feeding), and the presence of both diagnosed and undiagnosed diabetes. In acute heart failure (HF), hyperglycaemia has also been associated with mortality [3, 4]. Current guidelines for inpatient intervention for glucose are based on an absolute glucose value [5–7], but in AMI, HF, and AIS, the evidence to support these guidelines is lacking, and using an absolute value neglects the potential importance of the magnitude in change of glucose from baseline at presentation, and the direction of this change. The stress hyperglycaemia ratio (SHR), first described in 2015 [8], provides a measure of an individual’s acute hyperglycaemia, relative to their HbA1c. It is calculated by dividing the admission venous glucose by an estimated mean glucose taken from the HbA1c. An increasing body of evidence suggests that a raised SHR is associated with mortality, and may be a better discriminator than an individual’s absolute glucose value across a variety of medical conditions [9–11].

The aim of this meta analysis and systematic review is to synthesise the evidence assessing the association between the SHR and risk of all-cause mortality across the spectrum of three common cardiovascular presentations: AMI, AIS, and HF.

## Research design and methods

### Search strategy and study selection

The meta-analysis and systematic review were performed in accordance with the Cochrane Handbook for systematic reviews and PRISMA (Preferred Reporting Items for Systematic Reviews and Meta-Analyses) 2020 guideline. The protocol was registered on the PROSPERO database (PROSPERO 2023 CRD42023456421). The following databases were searched from the data of inception to 1st March 2024: Medline, Embase, Cochrane CENTRAL and Web of Science. Search terms are detailed in the supplementary material and were related to the index pathologies AMI, AIS, and HF, to the SHR, and relative hyperglycaemia, and mortality. Longitudinal studies (case control studies, cohort studies and randomised control trials) that reported risk ratios or odds ratios of all-cause mortality in relation to the SHR measured following admission to hospital for AMI, AIS or HF in humans > 18 years were included. Only studies measuring the SHR using an admission glucose level (or within first 24 hours) were included and those that used a fasting glucose were excluded (in keeping with the original definition of the SHR). Studies that evaluated the risk of all-cause mortality in relation to a composite of acute cardiovascular events that included AMI or AIS or HF, but were not exclusive to these conditions were included. Case reports, reviews, notes, meta-analyses, editorials, letter to the editor, commentaries, conference abstracts, and non-English studies were excluded. Studies which analysed data from participants with haemorrhagic stroke were excluded.

The effect of the SHR measurement on all-cause mortality was explored as a categorical variable. A high SHR was represented by the highest SHR category in each study, ranging from top half to top seventh SHR grouping across all studies. Studies that investigated the SHR as a continuous variable were included in the systematic review. Studies which analysed risk of mortality following haemorrhagic stroke were excluded.

### Data analysis

Two reviewers (Harriet Esdaile, HE and Shaila Khan, SK) screened title and abstracts independently. The same authors then undertook full-text evaluation. Where appropriate, the decision to include or exclude a record at any stage of screening was discussed with a third reviewer, who was the ultimate adjudicator. The online Covidence tool was used for the first phase screening. For each study the following data were extracted using a structured data extraction document: authors, year, source of data, pathology, recruitment dates, inclusion and exclusion criteria, follow up assessment and primary outcome ascertainment, timing of follow up, SHR calculation details, SHR categories, age, and sex of participants, number of participants with diabetes, risk ratio(s) for all-cause mortality, and co-variates used for the latter’s adjustment (Supplement Tables 3 and 4).

The Newcastle Ottawa Scale (NOS) for cohort studies was used to assess the quality of studies. Three domains (selection, comparability, exposure) were evaluated to provide a score ranging between 0 and 9. A score of ≥ 7 was deemed to indicate high quality, 4–6 moderate quality and < 3 poor quality. Two authors independently assessed the studies, and any disagreement between the authors was resolved with involvement of a third author.

Analyses were performed using STATA software (BE 17). Adjusted hazard or odds ratios for all-cause mortality for the highest SHR category for each study were pooled using the DerSimonian-Laird random effects model. A two-tailed p value less than 0.05 was considered statistically significant. Where studies reported subgroup risk ratios for participants with and without diabetes, with no overall estimate, the groups were included as separate estimates in the overall analysis. Similarly where an overall risk ratio was provided and a diabetes specific one, the overall risk ratio was used for the primary analysis. Studies that reported all-cause mortality as part of composite outcome were included. Standard errors were calculated using Cochrane meta analysis methodology. The I^2^ value was used to explore percentage of total variability that was due to between study heterogeneity and tested with Cochran’s Q test. I^2^ was assessed as: 0–25% (unimportant); 26–50% (moderate heterogeneity); 51–75% (substantial heterogeneity); and >75% (considerable heterogeneity). Publication bias was evaluated using a funnel plot, Egger’s test, and the trim and fill method.

Pre-specified subgroup analysis of participants with and without diabetes, and further analyses stratified by length of study follow up, and by reporting of all-cause mortality as a single outcome or as part of a composite outcome were conducted. Sensitivity analysis restricted to studies graded as of high quality was undertaken. Meta-regression using a random effects model with weighted age as a continuous co-variate (age weighted to age and proportion of participants in each SHR category per study) was performed.

### Data and resource availability

The datasets generated during and/or analysed in the current study are available from the corresponding author upon reasonable request.

## Results

**Fig. 1:**
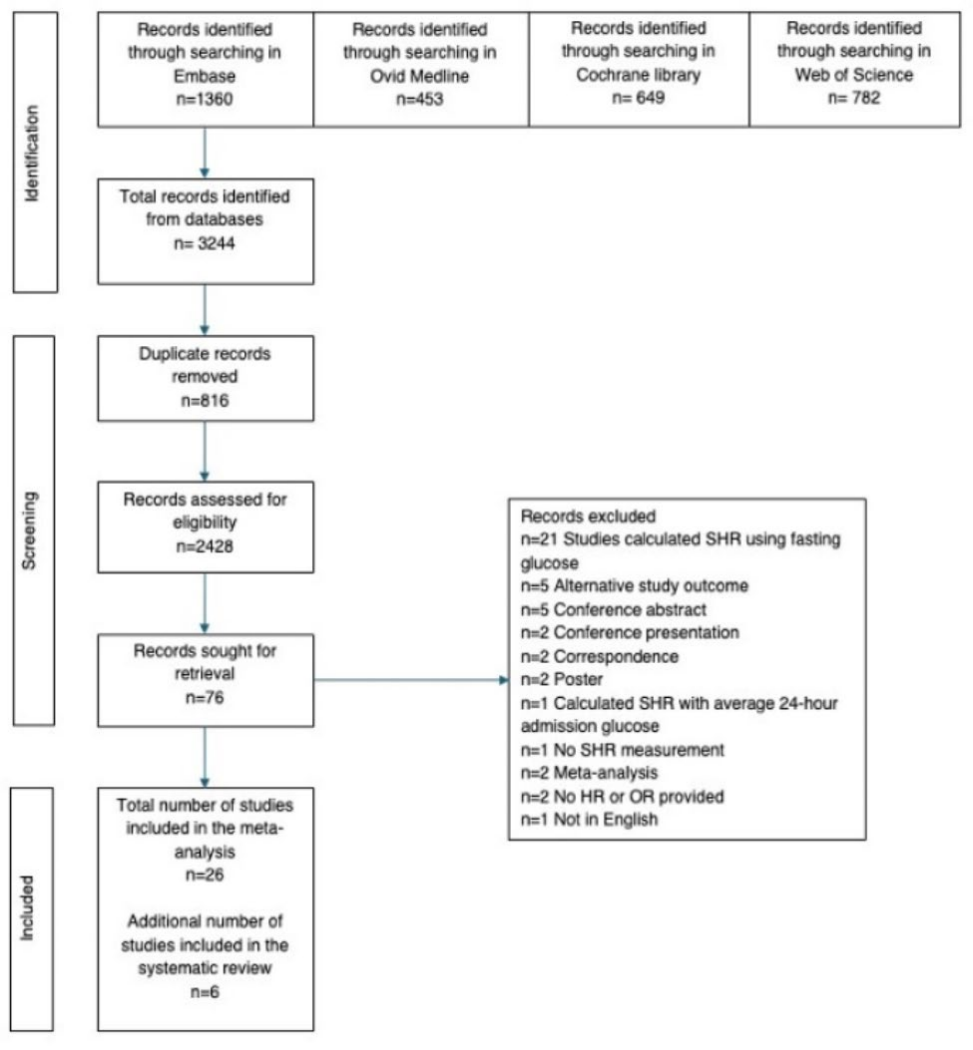
PRISMA diagram

In total 3,244 records were identified from database searches, of which 816 duplicate records were removed. Seventy-six records were selected for full text evaluation of which 26 were included in the meta-analysis, and an additional six records, which investigated SHR as a continuous variable, were included in the systematic review. The PRISMA flow diagram is shown in Fig. 1. The breakdown of studies included cohorts with HF (*n* = 6), AIS (*n* = 6) and AMI (*n* = 20). Sixteen studies assessed mortality as a single outcome, and 10 studies assessed it as part of a composite outcome. Characterisation of the SHR categories used in each study is detailed in Table 1. Four studies did not provide details of the SHR measurements used to stratify into categories.

**Table 1:** Characteristics of included studies

First author	Year	Study type	Data sources	Pathology	SHR categories	Cut off for higher SHR	Follow up	Total participants	Diabetes %	Male sex %	Age of total cohort (mean/median years)	Outcomes	Mortality as single or composite outcome
Carrera [48]	2021	Prospective cohort	Cardiology Department, Hospital del Mar, Spain	HF	Tertiles	Tertile 3 (high) Tertile 2 Tertile 1 (ref)	4 years	1062	55.6	56.7	72.60	All-cause mortality	Single
Cunha et al. [43]	2023	Retrospective cohort	Centro Hospitalar Universitario Sao Joao Porto	HF	Tertiles	> 1.16	3 months	599	51	44.1	76.00	All-cause mortality	Single
Mohammed et al. [49]	2024	Retrospective cohort	Shanghai Tenth People’s Hospital, China	HF	Halves	> 0.99	41 months (mean)	400	41.5	43.5	71.00	Composite outcome: all-cause mortality (death resulting from any reason, encompassing cardiovascular reasons) and readmission for HF	Composite
Zhou et al. [50]	2023	Prospective cohort	Peking University Third Hospital, China	HF	Quintles	>1.14 (high) 0.90 < SHR ≤ 1.14 0.77 < SHR ≤ 0.90 0.64 < SHR ≤ 0.77 (ref) SHR ≤ 0.64	3 years	780	100	63.3	68.90	All-cause mortality	Single
Li et al. [51]	2024	Retrospective cohort	Medical Information Mart for Intensive Care (MIMIC-IV, version 2.0) database, USA	HF	Sevenths	≥ 1.75 (high) 1.50–1.74 1.25–1.49 1.00–1.24 0.75–0.99 (ref) 0.50–0.74 < 0.50	1 year	8268	56.7	56.4	72.40	Primary outcome was the occurrence of AKI during the hospitalisation period. Secondary outcomes encompassed in-hospital mortality and one-year mortality	Single
Zhou et al. [52]	2022	Retrospective cohort	WECODe (West China Electronic medical record Collaboration of DiabEtes), China	HF	Tertiles	1.09–4.45 (high) 0.79–1.08 (ref) 0.16–0.78	IP	2875	65.2	61.3	71.20	Composite cardiac events (the combination of death during hospitalisation, requiring cardiopulmonary resuscitation, cardiogenic shock, and new episode of acute heart failure after admission), major AKI and major systemic infection during follow-up duration	Composite
Chen et al. [53]	2022	Retrospective cohort	Third Affiliated Hospital of Wenzhou Medical University, China	AIS	Halves	> 1.35	3 months	230	33	62.2	not given	Primary outcome was poor functional outcome, defined as 3-month mRS scores 3–6; the secondary outcomes included ENI and 3-month all-cause mortality.	Single
Peng et al. [54]	2023	Post hoc analysis of the RESCUE BT randomised controlled trial	55 stroke centres, China	Acute ischaemic stroke due to anterior circulation large vessel occlusion	Tertiles	≥ 1.30 (high) 1.08–1.29 ≤ 1.07 (ref))	90 days	542	27,7	56.5	68.00	The primary outcome was the proportion of favorable functional outcome defined as an mRS score of 2 points or less at 90 days. Secondary outcomes included a 90day mRS score of 0–1 and a 90day distribution of mRS scores. Safety outcomes included 90day mortality, the risk of sICH and ICH within 48 hours	Single
Wang et al. [55]	2019	Retrospective cohort	Endovascular Treatment for Acute Anterior Circulation Ischaemic stroke registry, 21 stroke cetners in China	AIS	Tertiles	Tertile 3 (high) Tertile 2 (ref) Tertile 1	90 days	321	25,6	61.1	not given	All-cause mortality	Single
Peng et al. [56]	2024	Retrospective cohort taken from the BASILAR study	47 stroke centres, China	Acute basilar artery occlusion	Tertiles	≥ 1.37 (high) 1.12–1.36 ≤ 1.11 (ref)	1 year	250	25.2	75.2	65.00	The primary efficacy outcome was a favorable outcome at 90 days, defined as an mRS score of 0–3	Single
Roberts et al. [46]	2021	Prospective cohort	Not stated	AIS	Halves	≥ 1.14	IP	300	30	53	not given	Composite of IP mortality, stroke exacerbation during hospitalization, discharge to a permanently higher level of care, or functional deficit at hospital discharge compared to admission	Composite
Shen et al. [57]	2021	Prospective observational	First Affiliated Hospital of Wenzhou Medical University, China	AIS	Continuous	n/a	3 months	341	22.6	71	66.4	Primary outcome was mRS score of 3–6 at the 3month follow-up appointment. Secondary outcomes included ENI, death within 3 months of follow-up, and intracerebral hemorrhage.	Single
Cui et al. [58]	2022	Prospective cohort	108 centres, China	STEMIand NSTEMI	Halves	Diabetes ≥ 1.20 No diabetes ≥ 1.08	2 years	6892	401	76	not given	All-cause mortality	Single
Kojima et al. [59]	2020	Prospective cohort	25 centres, Japan	STEMI	Halves	not given	5 years	6287	38.3	77.2	not given	All-cause mortality and admission due to heart failure	Single
Sia et al. [60]	2021	Retrospective cohort	Singapore Myocardial Infarction Registry, Singapore	STEMIand NSTEMI	Halves	Diabetes: STEMI > 1.68, NSTEMI > 1.53 No diabetes: STEMI > 1.51 NSTEMI > 1.27	1 year	9946	51.9	81.7	not given	All-cause mortality	Single
Xu et al. [61]	2022	Prospective cohort	247 centres, China	STEMI	Halves	≥ 1.329	30 days	5417	24.7	69.7	65.00	All-cause mortality and MACE	Single
Xu et al. [62]	2022	Retrospective cohort	Beijing Hospital, China	Acute coronary syndrome	Tertiles	≥ 0.832 (high) 0.725 ≤ SHR < 0.832 < 0.725 (ref)	IP	8196	53.8	64.3	68.00	All-cause mortality	Single
Zeng et al. [63]	2023	Post hoc analysis of a large prospective observational nationwide cohort study	Fuwai hospital (National Center for Cardiovascular Disease) and eight other medical centers throughout China.	AMI	Tertiles	> 1.10 (high) 0.84 < SHR ≤ 1.10 ≤ 0.84 (ref)	2 years	7226	44	82.3	not given	Primary outcome was MACE, a composite of all-cause mortality, MI, and unplanned revascularisation. Secondary outcome included individual components of the primary end point	Single
Xie et al. [64]	2023	Retrospective cohort	CRUISE-R study (Coronary Revascularisation in Patients On Dialysis, China	AMI	Tertiles	> 1.10 (high) 0.79 < SHR ≤ 1.10 (ref) ≤ 0.79	Maximum 7 years	714	63	75	62.00	The primary outcome was MACE, and the secondary outcomes were all-cause mortality and cardiovascular mortality	Single
Liu et al. [65]	2023	Retrospective cohort	Medical Information Mart for Intensive Care (MIMIC-IV, version 2.0) database, USA CIN- II (Cardiorenal ImprovemeNT II) registry, China	AMI	Quartiles	MIMIC- IV cohort ≥ 1.30 (high) 1.04–1.30 0.88-1.04 (ref) < 0.88 CIN-II cohort ≥ 1.23 (high) 1.02–1.23 0.86-1.02 (ref) < 0.86	MIMIC-IV: maximum of 12.1 years and CIN- II: 14.2 years maximum	4337 (MIMIC-IV = 2166, CIN-II = 2171)	MIMIC-IV:44.1% and CIN-II: 42.2%	MIMIC-IV: 69 and CIN-II:79.4	MIMIC- IV cohort:69 CIN-II cohort: 62.9	All-cause mortality	Single
Abdu et al. [66]	2023	Prospective cohort	Shanghai Tenth People’s Hospital (Tongji University, Shanghai, China)	AMI	Tertiles	≥ 0.84 (high) 0.73–0.84 ≤ 0.73 (ref)	34 months (mean)	410	19.3	52.7	Not given	MACE: which includes cardiac death, heart failure, nonfatal MI, stroke, and angina rehospitalization	Composite
Gao et al. [67]	2023	Prospective cohort	Fuwai Hospital, China	AMI	Halves	≥ 1.17	3.5 years (median)	1179	10	73.5	Not given	MACE: all-cause death, nonfatal MI, nonfatal stroke, revascularisation, and hospitalisation for UA or HF	Composite
Marenzi et al. [68]	2018	Prospective cohort	Centro Cardiologico Monzino, Milan, Italy	AMI	Halves	≥ 1.3	IP	1553	26.9	74.1	Not given	Combination of in-hospital mortality, nonfatal acute pulmonary oedema, and cardiogenic shock	Composite
Lin et al. [69]	2023	Prospective cohort	Guangdong Provincial People’s Hospital, China	AMI	Tertiles	≥ 1.176 (high) 0.929 ≤ SHR < 1.176 < 0.929 (ref)	IP	2841	31.1	82.6	62.27	The primary outcome was the occurrence of pulmonary infection during hospitalization, and the secondary endpoint was in-hospital MACEs, composed of all-cause mortality, stroke, target vessel revascularisation, or recurrent myocardial infarction	Composite
Gao et al. [70]	2019	Prospective cohort	Beijing Friendship Hospital, China	AMI	Halves	≥ 1.32	IP	1300	43.9	77.9	Not given	Combination of the most clinically relevant haemodynamic consequences after STEMI, including in-hospital mortality, cardiogenic shock, and acute pulmonary oedema	Composite
Yang et al. [71]	2022	Prospective cohort	Fuwai Hospital, China	AMI	Quintiles	> 0.90 (high) 0.81 < SHR ≤ 0.90) 0.75 < SHR ≤ 0.81) (ref) 0.70 < SHR ≤ 0.75) ≤ 0.70	IP	5562	39.3	76.7	Not given	MACCE at the 2-year follow-up, including all-cause death, nonfatal MI, nonfatal stroke, and TVR	Composite
Yang et al. [72]	2017	Retrospective cohort	COnvergent REgistry of cAtholic and Chonnam University for Acute Myocardial Infarction (COREA-AMI) registry, Korea	AMI	Halves	not given	IP	2523	49.5	74.5	Not given	MACCE (a composite of all-cause death, nonfatal recurrent MI or nonfatal stroke)	Composite
Chen et al. [73]	2023	Retrospective cohort	11 hospitals in Chengdu, Sichuan, China	STEMI, NSTEMI	Continuous	n/a	IP	613	37.4	70.3	67.1	In-hospital mortality	Single
Gao et al. [74]	2021	Retrospective cohort	Cardiovascular Center Beijing Friendship Hospital Database (CBD BANK)	STEMI and NSTEMI	Continuous	n/a	IP	1215	100	68.1	72.4	Primary outcome was AKI, secondary outcomes included all- cause death and cardiogenic shcok during hospitalisation	Single
Xiong et al. [75]	2023	Prospective cohort	11 hospitals in Chengdu, Sichuan, China	STEMI,NSTEMI, UA	Continuous	n/a	Median 31.33 months	714	50.8	74.1	Not given	The primary outcome was AKI and secondary outcomesincluded all-cause death and cardiogenic shock during hospitalisation	Composite
Guo et al. [76]	2023	Retrospective cohort	China	STEMI	Continuous	n/a	IP	1944	40	77.4	58.43	In-hospital MACCE, defined as acute ischaemic stroke, mechanical complications of MI, cardiogenic shock, and all-cause death	Single
Wei et al. [77]	2023	Retrospective cohort	Not stated	STEMI	Continuous	n/a	IP	1099	27	62.6	62.55	The primary outcomes were in-hospital death and all-cause mortality following STEMI	Single

Abbreviations: AKI, acute kidney injury; ENI, early neurological improvement; ICH, intracranial haemorrhage; IP, inpatient; MACCE, Major adverse cardiac and cerebrovascular event; MACE, Major adverse cardiac event; MI, myocardial infarction; mRS modified Rankin Scale; NSTEMI, non-ST elevation myocardial infarction; ref, reference; STEMI, ST-elevation myocardial infarction; TVE, target vessel revascularisation; sICH, symptomatic intracranial haemorrhage

**Fig. 2:**
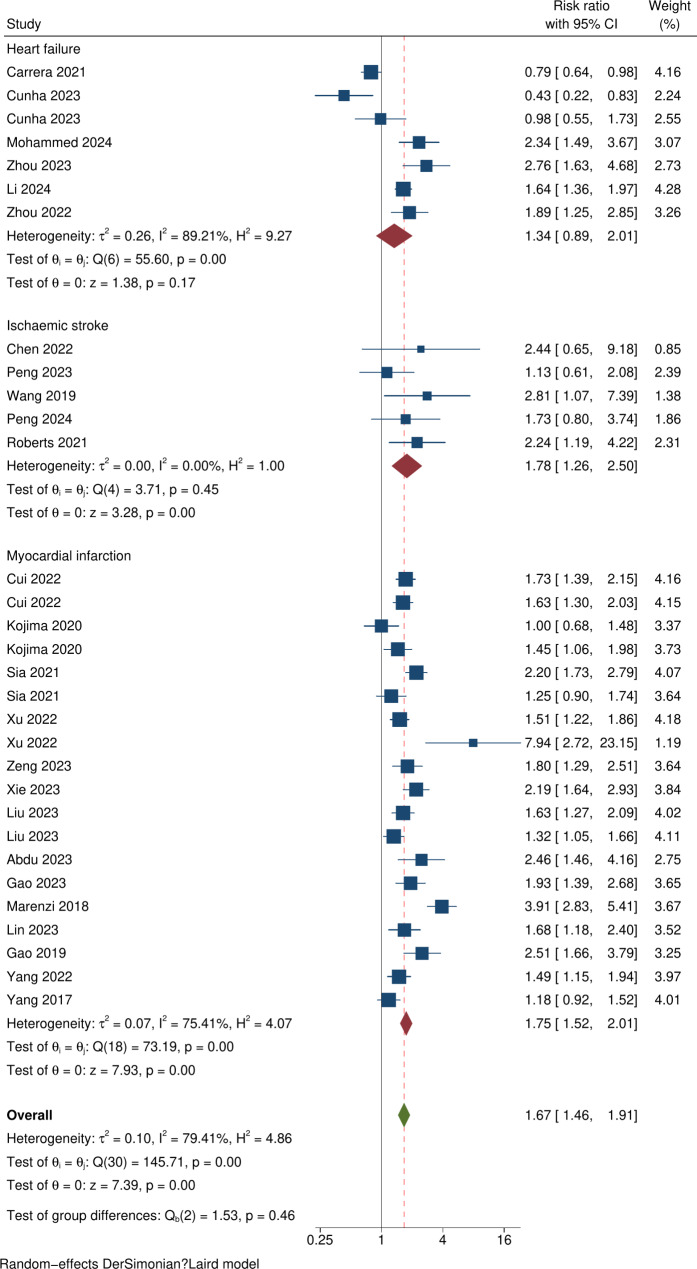
Forest plot of the prognostic impact of the SHR (higher vs. lower) and risk of all-cause mortality across pathologies

Thirty-one estimates from the 26 studies contributed to the meta-analysis (Fig. 2). Total participants numbered 80,010. Of these, 36,112 had a diagnosis of diabetes (45.1%) and 49,535 were male (62.6%). Age (measured as either mean or median) of the total cohorts for the studies ranged from 62 to 76 years. Table 1 shows the main characteristics of the included studies. Results of risk of bias using the NOS for cohort studies tool is found in the supplementary material (Table 2).

Participants experiencing an admission to hospital with cardiovascular disease and a raised SHR had a significantly higher risk ratio for all-cause mortality compared to those with a lower SHR (RR = 1.67 [95% CI 1.46–1.91]; p = < 0.001, I^2^ = 79.4%; p < 0.001, Fig. 2). Subgroup differences by index pathology were not identified (p = 0.46). Primary analysis of the HF cohort indicated a raised risk ratio, but the confidence interval for the HF cohort was wide and included the null, reflecting smaller sample sizes (RR 1.34 [95% CI 0.89–2.01]; p = 0.17, I^2^ = 89.2%). The highest heterogeneity was found in the HF studies. Following removal of the HF cohort in a sensitivity analysis the overall I^2^ was reduced to 70.3% with a risk ratio for the AMI and AIS cohorts of 1.75 [95% CI 1.54–1.99; p < 0.001] (Fig. 1, supplementary material).

**Fig. 3:**
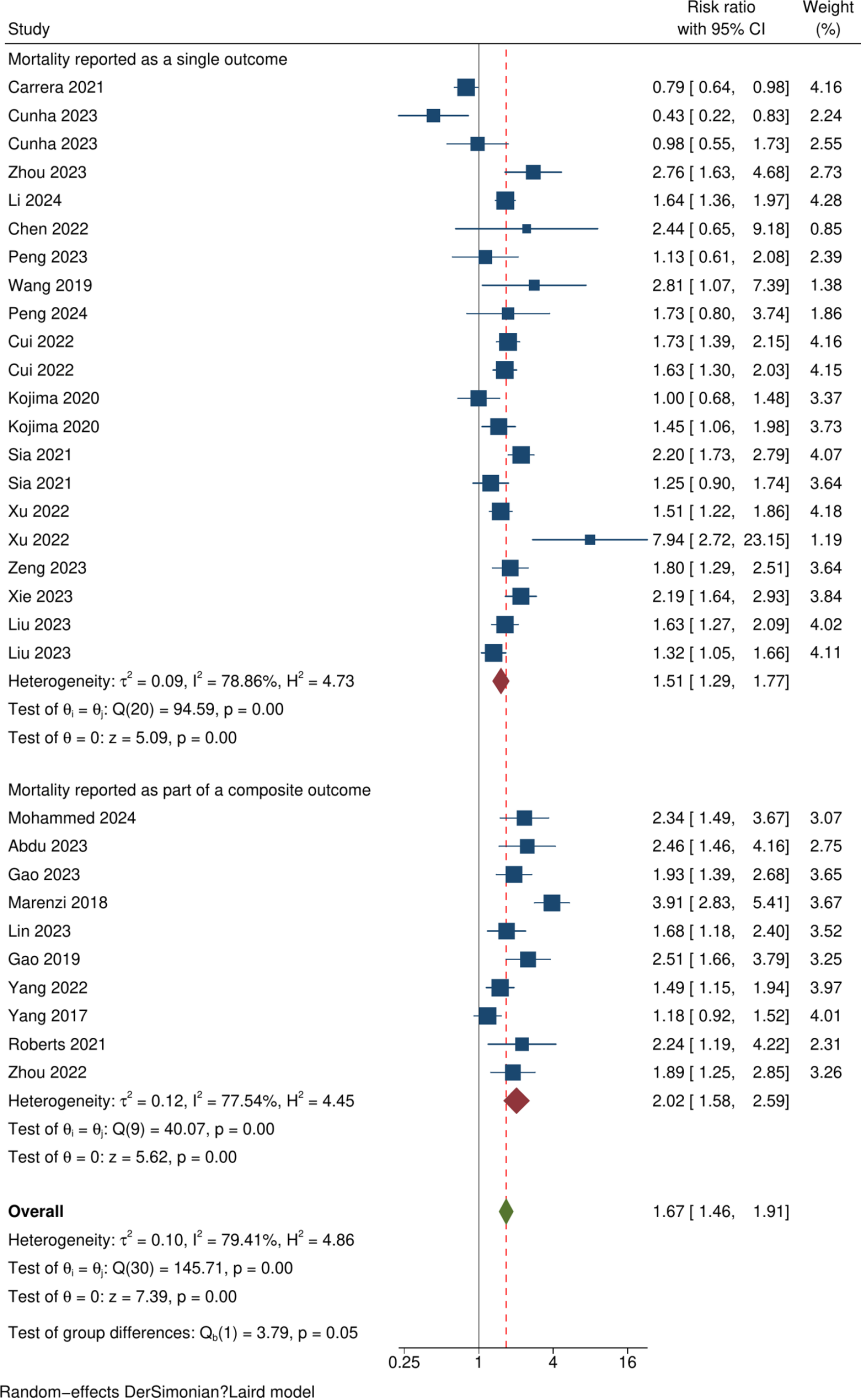
Forest plot showing subgroup analysis based on mortality measured as a single outcome or as part of a composite outcome

**Fig. 4:**
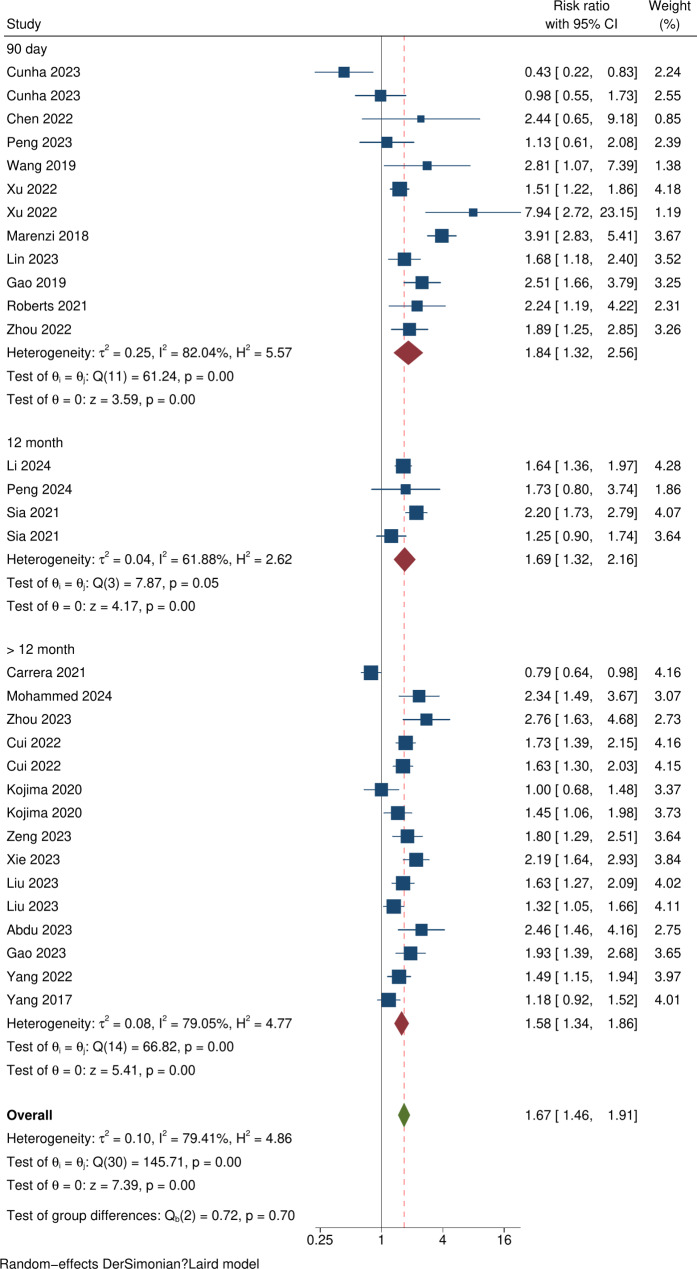
Forest plot showing subgroup analysis based on length of follow up

Analysis of studies reporting all-cause mortality as a single outcome identified a raised risk ratio (RR) of 1.51 ([95% CI 1.29–1.77]; p < 0.001, I^2^ = 78.86%; p = < 0.001) as did those that reported all-cause mortality as part of a composite outcome (RR 2.02 [95% CI 1.58–2.59]; p < 0.001, I^2^ = 77.54%; p < 0.001) with test group difference  p = 0.05 (Fig. 3). On subgroup analysis by length of follow up, higher SHR values were associated with increased risk of all-cause mortality at 90 day, 12 months, > 12 months of follow up, with RRs of 1.84 (95% CI 1.32–2.56), 1.69 (95% CI 1.32–2.16) and 1.58 (95% CI 1.34–1.86) respectively (Fig. 4). No between group difference was identified ( p = 0.70).

**Fig. 5:**
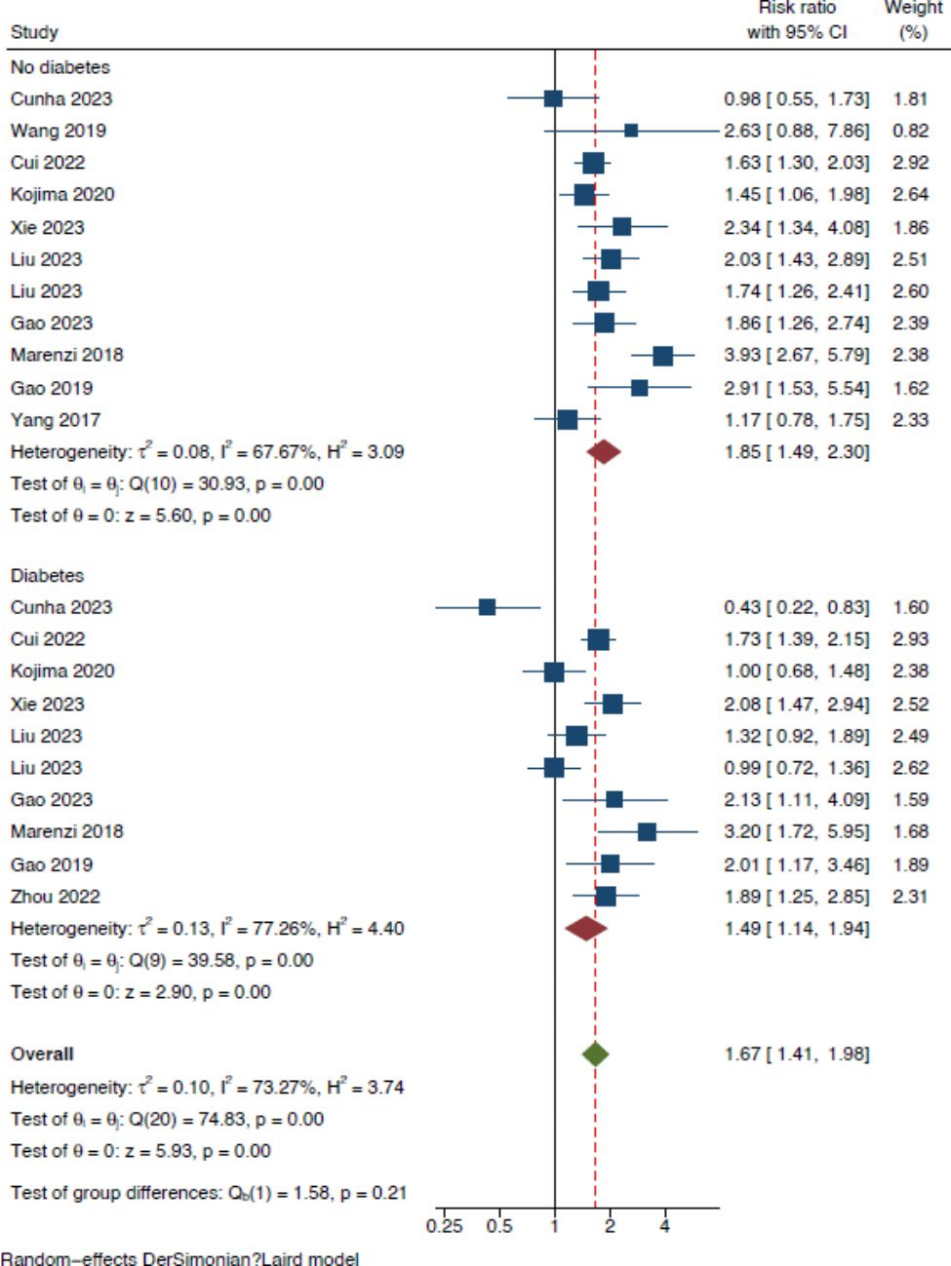
Forest plot showing subgroup analysis based on diabetes status

Analysis stratifying by diabetes status did not reveal significant group differences for participants with and without diabetes (RR = 1.49 [95% CI 1.14–1.94] for diabetes, and RR = 1.85 [95% CI 1.49–2.30] for no diabetes;  p = 0.21 for subgroup differences, Fig. 5). After removal of the 2 studies graded medium quality in a sensivity analysis, the risk ratio remained elevated at 1.66 [95% CI 1.47–1.87] (Fig. 2, supplementary material). We did not find strong evidence of publication bias (Egger’s test *p* = 0.14). We used the trim and fill method for publication bias to evaluate the change in the magnitude of the association following imputation. Six estimates were imputed providing an adjusted risk ratio of 1.49 [95% CI (1.29–1.72)].

On meta-regression with weighted age (age weighted to age and proportion of participants in each SHR category per study) as a continuous variable, we did not see any effect modification of the risk ratio (p = 0.8). A random effects weighted bubble plot is found in the supplementary material (Fig. 4).

Six papers (n = 5 AMI and n = 1 AIS) analysed the effect of the SHR on all-cause mortality when treated as a continuous variable. In the AMI cohort the SHR was significantly associated with all-cause mortality in all studies. The relationship was not significant for AIS (OR 2.992 [95% CI 0.372–24.049]).

## Discussion

This comprehensive meta-analysis assessed the influence of SHR on all-cause mortality in 80,010 people presenting across the spectrum of three common cardiovascular pathologies: AMI, AIS and HF. A higher SHR was associated with a higher risk ratio of all-cause mortality in AMI and AIS, with a trend towards a raised risk ratio in the HF cohort that was not significant. The association remained significant regardless of diabetes status, across studies that analysed mortality as a single outcome, or as part of a composite outcome, and across studies with variable length of follow up. Age was not identified as a mediator for the relationship.

The consistently reported relationship between hyperglycaemia and deleterious longer term outcomes following admission with an ischaemic event is not fully understood, and it remains unclear if hyperglycaemia is a causal risk factor. Glucose homeostasis is tightly controlled in humans and the stress response, whilst thought to be physiological, remains loosely defined as ‘the relative increase in glucose due to the inflammatory and neuro-hormonal derangements that occur during a major illness’ [8]. Hyperglycaemia at the time of admission with acute illness may have a plethora of negative effects, promoting oxidative stress [12], potentiating endothelial dysfunction and impairing vasodilatation [13, 14]. Hyperglycaemia may induce a pro-coagulant state [15] enhancing platelet reactivity [16] and driving alterations in plasmin’s fibrinolytic activity [17]. Following AIS, stimulation of both the tissue factor pathway and of the production of thrombin-antithrombin complexes diminish the activity of recombinant tissue plasminogen activator, used in thrombolysis, in both animal models and in clinical practice [18, 19]. Hyperglycaemia is associated with several aspects of infarct evolution: reduced perfusion, impaired recanalisation, reperfusion injury and intracranial haemorrhage [20, 21].

Shear stress-induced platelet activation is enhanced in acute hyperglycaemia [22] and in combination with other pro-inflammatory stimuli, could amplify myocardial necrosis at the peri-infarct region following AMI. In one study in people experiencing a STEMI with glucose > 10mmol/L who received tight glycaemic control, both the number and differentiation of endothelial progenitor cells was increased compared to controls, and this was hypothesised to be responsible for an improvement in the myocardial salvage score (an assessment of the amount of salvaged myocardium) measured up to 180 days post infarct compared to controls who did not receive such control [23]. Hyperglycaemia is associated with the no flow phenomenon following AMI [24, 25], and is also associated with prolongation of the QT interval [26], making the myocardium susceptible to ventricular arrythmias.

The relationship identified in this meta-analysis between a higher SHR and all-cause mortality suggests that the magnitude of the change of an individual’s glucose from their background glucose at the time of presentation is of prognostic importance. Furthermore whilst studies suggest that hyperglycaemia at admission holds a greater association with mortality for those without diabetes [2, 8, 27], the SHR measurement is associated with mortality irrespective of diabetes status [8, 28].

The results of several large interventional trials investigating hyperglycaemia at presentation with AIS and AMI are mixed [29–33], and are lacking for HF. Choosing an absolute glucose value on which to intervene and monitor may have led to mixed cohort and RCT study populations where true stress hyperglycaemia is mixed in with suboptimally controlled diabetes. The Stroke Hyperglycaemia Insulin Network Effect (SHINE) randomised trial [29] attempted to circumvent this challenge by using differing cut off glucose values for recruitment: > 6.1mmol/L for those with known diabetes, and > 8.3mmol/L for those without diabetes. The Diabetes Mellitus Insulin-Glucose Infusion in Acute Myocardial Infarction (DIGAMI)1 trial, the only trial to show a mortality benefit related to glycaemic control post AMI, however recruited people with or without known diabetes and glucose > 11mmol/L peri-AMI, and the subsequent DIGAMI 2 trial enrolled participants with known diabetes, or glucose > 11mmol/L.

If an admission glucose is higher than the estimated mean glucose, the SHR is > 1.0 suggesting a relative hyperglycaemia, and conversely is < 1.0 when the admission glucose is lower than the estimated mean glucose, suggesting a relative hypoglycaemia. However it is noteworthy that no universal definition for a high, low or, reference range for the SHR exists. The SHR cut off values of each study in this analysis were unique and referenced to their own population, either by a receiver operating characteristic (ROC) analysis, or by pre-specified division of the population into equal categories using SHR measurement, with the threshold value for the high SHR corresponding to the cut off for the highest category, and a lower, or lowest category being chosen as a reference for comparison. SHR threshold values varied for both the high and low categories across studies: 22 studies had a threshold value for a high SHR category > 1, and approximately half had a low or reference range with SHR values exclusively < 1. One interpretation of our results therefore is that a raised SHR is deleterious, and may be more damaging than a relative hypoglycaemia.

Hypoglycaemia is detrimental in the context of hospitalisation for cardiovascular disease [34–36] and in the critical care setting [37]. Whilst the concept of relative hypoglycaemia has been identified [38], it has not been adopted clinically, and little is known about its potential effects on mortality in cardiovascular disease. Graded hypoglycaemic clamp studies [39, 40] have identified an increase in the glycaemic threshold for autonomic symptoms in people with type 2 diabetes that are modified by changes in background diabetes control, and work from the critical care setting has identified that a relative hypoglycemia of > 30% of expected glucose derived from HbA1c is associated with mortality [41, 42], regardless of the absolute hypoglycaemia burden defined as glucose < 3.9mmol/L. Of the two HF studies in this analysis reporting a protective effect of a raised SHR, one did not report their SHR category thresholds and the other used a threshold of > 1.16 for a high SHR and a low SHR category cut of < 0.88 [43]. They reported a significantly raised HR of all-cause mortality for only those in the low SHR category and diabetes (HR 2.34 [95% CI 1.25–4.38] for those with diabetes and HR 1.02 [95% CI 0.58–1.81] for those without diabetes). Whether a relative hypoglycaemia is associated with mortality, at what threshold, and the potential mediation of any relationship by presence of diabetes, needs further investigation.

The pooled estimate from the studies that analysed participants admitted with decompensated heart failure did not show a significant association with all-cause mortality. This may have been due to the significant heterogeneity seen across studies assessing these conditions (I^2^ of 89.21%). One contributing factor to this heterogeneity may have been differences in case ascertainment, with differing definitions for acute HF used across studies. Alternatively, experiencing an episode of decompensated cardiac failure may provoke a different stress response from an AMI and AIS, and this may be dependent on the severity of circulatory compromise, and background function of the myocardium. We were not able to assess these parameters.

In those with acute heart failure multi-morbidity is highly prevalent [44, 45]. The contribution of pre-existing co-morbidities in potentially mediating the stress response, and potentially the SHR measurement, as well as outcomes, must be acknowledged. All-cause mortality captured in the longer term will be increasingly associated with any polymorbidity. Baseline adjustment for concurrent co-morbidities across the heart failure studies was variable. For those experiencing an ischaemic stroke there is an independent effect on outcome related to pre-existing morbidity [46]. Notably, the two HF studies that showed a protective effect of a raised SHR were from two Iberian cohorts (one Portuguese and one Spanish), and the four that showed increased risk of mortality with increased SHR were derived from three Chinese and one American cohort. Ethnic, and population differences, may drive risk of mortality.

There was significant heterogeneity identified in our meta-analysis and therefore our conclusions should be interpreted with caution. Unexplained heterogeneity in our analysis may originate from the variable SHR cut offs across the studies, variable definitions of the index pathology, underlying differences in the populations studied, and differing study level adjustment for other variables when assessing the relationship between the SHR and all-cause mortality. An individual participant data meta-analysis may address some of this heterogeneity. Future research should aim to establish standardised thresholds for the SHR, to facilitate potential clinical application as a prognostic marker and could investigate whether SHR may be a therapeutic target. Studies that utilised ROC to identify an SHR threshold provide extra insight, but further analysis of such studies is only possible with individual participant level data.

We used observational data, and causality cannot be established. Lack of randomisation or matching may result in confounding and for retrospective studies, and there may be bias in which participants had an HbA1c available when sampling. Additionally, whilst all studies included used a glucose value taken at admission, or within 24 hours of admission, the method used to calculate estimated average glucose for the denominator in the ratio differed across studies and may have inadvertently affected the accuracy of the measurement. This study did not undertake analysis by detailed subtype of pathology such as NSTEMI and STEMI as studies tended to group these together. Additionally we did not undertake analysis by presence or absence of obstruction of coronary arteries (myocardial infarction with nonobstructive coronary arteries - MINOCA). MINOCA has a reported prevalence of 3.5–15% in myocardial infarction [47]. Treatment modality was not included in adjustment for risk ratios across all studies.

## Conclusion

Our findings suggest that an elevated SHR is associated with risk of all-cause mortality in people admitted to hospital with an acute myocardial infarction or acute ischaemic stroke. Whilst a similar trend was seen for people admitted with heart failure, this was not significant. Future work is required to characterise further the SHR measurement, to investigate the impact of relative hypoglycaemia, to evaluate any potential for the inclusion of the SHR measurement in cardiovascular risk stratification, and to investigate the SHR as a therapeutic target.

## Electronic supplementary material

Below is the link to the electronic supplementary material.

Supplementary Material 1

## Data Availability

The datasets generated during and/or analysed in the current study are available from the corresponding author upon reasonable request.

## Abbreviations

AIS: Acute ischaemic stroke
AMI: Acute myocardial infarction
HF: Heart failure
MINOCA: Myocardial infarction with nonobstructive coronary arteries
PRISMA: Preferred reporting Items for systematic reviews and meta-analyses
RR: Relative risk
SHR: Stress hyperglcyaemia ratio
