# Supplementary material for: The association between the stress hyperglycaemia ratio and mortality in cardiovascular disease: a meta-analysis and systematic review
Source: Cardiovasc Diabetol. 2024 Nov 16;23:412. doi: 10.1186/s12933-024-02454-1 (PMC11568630; doi:10.1186/s12933-024-02454-1)
Supplement: Supplementary file 1 — Supplementary Material 1 [file 12933_2024_2454_MOESM1_ESM.docx]

**Supplementary material**

|  | | Page |
| --- | --- | --- |
| Search strategies | 2 | |
| Table 1: List of studies excluded at the full text screening stage | 12 | |
| Table 2: Quality assessment (Newcastle-Ottawa quality assessment scale for cohort studies)  Table 3: Variables used in adjustment of risk ratios in each study | 17 | |
| Figure 1: Forest plot with HF studies removed | 24 | |
| Figure 2: Forest plot with low/medium quality studies removed | 25 | |
| Figure 3: Contour enhanced funnel plot | 26 | |
| Figure 4: Meta regression for age bubble plot | 26 | |
| Table 4: Data extraction form | 27 | |
| Table 5: PRISMA 2020 Checklist | 29 | |
|  |  | |
|  |  | |

**Search strategies**

**Embase <1974 to 2024 March 01>**

1 exp heart infarction/ 456983

2 coronary thrombosis.mp. or exp coronary artery thrombosis/ 9277

3 acute coronary.mp. [mp=title, abstract, heading word, drug trade name, original title, device manufacturer, drug manufacturer, device trade name, keyword heading word, floating subheading word, candidate term word] 101427

4 exp unstable angina pectoris/ 28205

5 myocardial infarct*.mp. [mp=title, abstract, heading word, drug trade name, original title, device manufacturer, drug manufacturer, device trade name, keyword heading word, floating subheading word, candidate term word] 361549

6 heart infarct.mp. [mp=title, abstract, heading word, drug trade name, original title, device manufacturer, drug manufacturer, device trade name, keyword heading word, floating subheading word, candidate term word] 286

7 cardiac infarct.mp. [mp=title, abstract, heading word, drug trade name, original title, device manufacturer, drug manufacturer, device trade name, keyword heading word, floating subheading word, candidate term word] 295

8 heart attack.mp. [mp=title, abstract, heading word, drug trade name, original title, device manufacturer, drug manufacturer, device trade name, keyword heading word, floating subheading word, candidate term word] 8037

9 heart muscle infarction.mp. [mp=title, abstract, heading word, drug trade name, original title, device manufacturer, drug manufacturer, device trade name, keyword heading word, floating subheading word, candidate term word] 4

10 myocardium infarct*.mp. [mp=title, abstract, heading word, drug trade name, original title, device manufacturer, drug manufacturer, device trade name, keyword heading word, floating subheading word, candidate term word] 565

11 subendocardial infarct*.mp. [mp=title, abstract, heading word, drug trade name, original title, device manufacturer, drug manufacturer, device trade name, keyword heading word, floating subheading word, candidate term word] 397

12 transmural cardiac infarct*.mp. [mp=title, abstract, heading word, drug trade name, original title, device manufacturer, drug manufacturer, device trade name, keyword heading word, floating subheading word, candidate term word] 7

13 transmural heart infarct*.mp. [mp=title, abstract, heading word, drug trade name, original title, device manufacturer, drug manufacturer, device trade name, keyword heading word, floating subheading word, candidate term word] 2

14 acs.mp. [mp=title, abstract, heading word, drug trade name, original title, device manufacturer, drug manufacturer, device trade name, keyword heading word, floating subheading word, candidate term word] 57624

15 ami.mp. [mp=title, abstract, heading word, drug trade name, original title, device manufacturer, drug manufacturer, device trade name, keyword heading word, floating subheading word, candidate term word] 39865

16 (coronary adj3 syndrome$).mp. [mp=title, abstract, heading word, drug trade name, original title, device manufacturer, drug manufacturer, device trade name, keyword heading word, floating subheading word, candidate term word] 99565

17 (unstable adj3 angina).mp. [mp=title, abstract, heading word, drug trade name, original title, device manufacturer, drug manufacturer, device trade name, keyword heading word, floating subheading word, candidate term word] 34157

18 1 or 2 or 3 or 4 or 5 or 6 or 7 or 8 or 9 or 10 or 11 or 12 or 13 or 14 or 15 or 16 or 17 601926

19 brain infarction/ or brain stem infarction/ or cerebellum infarction/ 68794

20 exp brain ischemia/ 220181

21 carotid artery disease/ 10976

22 exp carotid artery obstruction/ 11887

23 cerebral artery disease/ 5791

24 exp cerebrovascular accident/ 330012

25 exp occlusive cerebrovascular disease/ 52856

26 stroke patient/ 43199

27 ((brain or cerebr$ or cerebell$ or vertebrobasil$ or hemispher$ or intracran$ or intracerebral or infratentorial or supratentorial or middle cerebr$ or mca$ or anterior circulation or basilar artery or vertebral artery) adj5 (isch?emi$ or infarct$ or thrombo$ or emboli$ or occlus$ or hypoxi$)).mp. [mp=title, abstract, heading word, drug trade name, original title, device manufacturer, drug manufacturer, device trade name, keyword heading word, floating subheading word, candidate term word] 346343

28 19 or 20 or 21 or 22 or 23 or 24 or 25 or 26 or 27 655436

29 heart failure/ or acute heart failure/ or cardiogenic shock/ or exp congestive heart failure/ or diastolic dysfunction/ or forward heart failure/ or high output heart failure/ or systolic dysfunction/ 495123

30 congestive cardiomyopathy/ 37290

31 exp heart ventricle failure/ 59047

32 ((heart or cardiac or myocardial) adj2 (failure or decompensation)).mp. [mp=title, abstract, heading word, drug trade name, original title, device manufacturer, drug manufacturer, device trade name, keyword heading word, floating subheading word, candidate term word] 533167

33 ((congestive or acute or decompensat*) adj2 heart failure).mp. [mp=title, abstract, heading word, drug trade name, original title, device manufacturer, drug manufacturer, device trade name, keyword heading word, floating subheading word, candidate term word] 144429

34 ((dilated or congestive) adj2 cardiomyopath*).mp. [mp=title, abstract, heading word, drug trade name, original title, device manufacturer, drug manufacturer, device trade name, keyword heading word, floating subheading word, candidate term word] 49250

35 cardiogenic shock.mp. [mp=title, abstract, heading word, drug trade name, original title, device manufacturer, drug manufacturer, device trade name, keyword heading word, floating subheading word, candidate term word] 42989

36 ((ventricular or ventricle*) adj2 (failure or insufficien* or dysfunction*)).mp. [mp=title, abstract, heading word, drug trade name, original title, device manufacturer, drug manufacturer, device trade name, keyword heading word, floating subheading word, candidate term word] 94763

37 lvsd.mp. [mp=title, abstract, heading word, drug trade name, original title, device manufacturer, drug manufacturer, device trade name, keyword heading word, floating subheading word, candidate term word] 1624

38 29 or 30 or 31 or 32 or 33 or 34 or 35 or 36 or 37 651395

39 exp hyperglycemia/ 123445

40 hyperglyc?emi*.mp. [mp=title, abstract, heading word, drug trade name, original title, device manufacturer, drug manufacturer, device trade name, keyword heading word, floating subheading word, candidate term word] 163104

41 glyc?emi*.mp. [mp=title, abstract, heading word, drug trade name, original title, device manufacturer, drug manufacturer, device trade name, keyword heading word, floating subheading word, candidate term word] 149052

42 elevated glucose.mp. [mp=title, abstract, heading word, drug trade name, original title, device manufacturer, drug manufacturer, device trade name, keyword heading word, floating subheading word, candidate term word] 3216

43 39 or 40 or 41 or 42 284902

44 stress hyperglyc?emi* ratio.mp. [mp=title, abstract, heading word, drug trade name, original title, device manufacturer, drug manufacturer, device trade name, keyword heading word, floating subheading word, candidate term word] 138

45 ((hyperglyc?emi* or glyc?emi*) adj2 ratio).mp. [mp=title, abstract, heading word, drug trade name, original title, device manufacturer, drug manufacturer, device trade name, keyword heading word, floating subheading word, candidate term word] 395

46 ratio.mp. [mp=title, abstract, heading word, drug trade name, original title, device manufacturer, drug manufacturer, device trade name, keyword heading word, floating subheading word, candidate term word] 2050581

47 stress.mp. [mp=title, abstract, heading word, drug trade name, original title, device manufacturer, drug manufacturer, device trade name, keyword heading word, floating subheading word, candidate term word] 1674856

48 relative hyperglyc?emi*.mp. [mp=title, abstract, heading word, drug trade name, original title, device manufacturer, drug manufacturer, device trade name, keyword heading word, floating subheading word, candidate term word] 73

49 relative hyperglyc?emi*.mp. [mp=title, abstract, heading word, drug trade name, original title, device manufacturer, drug manufacturer, device trade name, keyword heading word, floating subheading word, candidate term word] 73

50 relative.mp. [mp=title, abstract, heading word, drug trade name, original title, device manufacturer, drug manufacturer, device trade name, keyword heading word, floating subheading word, candidate term word] 1295023

51 43 and 50 11255

52 43 and 46 and 47 1937

53 44 or 45 or 48 or 49 or 51 or 52 13297

54 18 or 28 or 38 1655487

55 53 and 54 1360

**Ovid MEDLINE(R) ALL <1946 to March 01, 2024>**

1 exp Myocardial Infarction/ 196703

2 exp Coronary Thrombosis/ 8379

3 acute coronary.mp. [mp=title, book title, abstract, original title, name of substance word, subject heading word, floating sub-heading word, keyword heading word, organism supplementary concept word, protocol supplementary concept word, rare disease supplementary concept word, unique identifier, synonyms, population supplementary concept word, anatomy supplementary concept word] 49936

4 exp Angina, Unstable/ 11375

5 myocardial infarct*.mp. [mp=title, book title, abstract, original title, name of substance word, subject heading word, floating sub-heading word, keyword heading word, organism supplementary concept word, protocol supplementary concept word, rare disease supplementary concept word, unique identifier, synonyms, population supplementary concept word, anatomy supplementary concept word] 286010

6 heart infarct.mp. [mp=title, book title, abstract, original title, name of substance word, subject heading word, floating sub-heading word, keyword heading word, organism supplementary concept word, protocol supplementary concept word, rare disease supplementary concept word, unique identifier, synonyms, population supplementary concept word, anatomy supplementary concept word] 523

7 cardiac infarct.mp. [mp=title, book title, abstract, original title, name of substance word, subject heading word, floating sub-heading word, keyword heading word, organism supplementary concept word, protocol supplementary concept word, rare disease supplementary concept word, unique identifier, synonyms, population supplementary concept word, anatomy supplementary concept word] 270

8 heart attack.mp. [mp=title, book title, abstract, original title, name of substance word, subject heading word, floating sub-heading word, keyword heading word, organism supplementary concept word, protocol supplementary concept word, rare disease supplementary concept word, unique identifier, synonyms, population supplementary concept word, anatomy supplementary concept word] 5374

9 heart muscle infarction.mp. [mp=title, book title, abstract, original title, name of substance word, subject heading word, floating sub-heading word, keyword heading word, organism supplementary concept word, protocol supplementary concept word, rare disease supplementary concept word, unique identifier, synonyms, population supplementary concept word, anatomy supplementary concept word] 1

10 myocardium infarct*.mp. [mp=title, book title, abstract, original title, name of substance word, subject heading word, floating sub-heading word, keyword heading word, organism supplementary concept word, protocol supplementary concept word, rare disease supplementary concept word, unique identifier, synonyms, population supplementary concept word, anatomy supplementary concept word] 328

11 subendocardial infarct*.mp. [mp=title, book title, abstract, original title, name of substance word, subject heading word, floating sub-heading word, keyword heading word, organism supplementary concept word, protocol supplementary concept word, rare disease supplementary concept word, unique identifier, synonyms, population supplementary concept word, anatomy supplementary concept word] 309

12 transmural cardiac infarction.mp. [mp=title, book title, abstract, original title, name of substance word, subject heading word, floating sub-heading word, keyword heading word, organism supplementary concept word, protocol supplementary concept word, rare disease supplementary concept word, unique identifier, synonyms, population supplementary concept word, anatomy supplementary concept word] 3

13 transmural heart infarction.mp. [mp=title, book title, abstract, original title, name of substance word, subject heading word, floating sub-heading word, keyword heading word, organism supplementary concept word, protocol supplementary concept word, rare disease supplementary concept word, unique identifier, synonyms, population supplementary concept word, anatomy supplementary concept word] 1

14 acs.mp. [mp=title, book title, abstract, original title, name of substance word, subject heading word, floating sub-heading word, keyword heading word, organism supplementary concept word, protocol supplementary concept word, rare disease supplementary concept word, unique identifier, synonyms, population supplementary concept word, anatomy supplementary concept word] 30563

15 ami.mp. [mp=title, book title, abstract, original title, name of substance word, subject heading word, floating sub-heading word, keyword heading word, organism supplementary concept word, protocol supplementary concept word, rare disease supplementary concept word, unique identifier, synonyms, population supplementary concept word, anatomy supplementary concept word] 24072

16 (coronary adj3 syndrome$).mp. [mp=title, book title, abstract, original title, name of substance word, subject heading word, floating sub-heading word, keyword heading word, organism supplementary concept word, protocol supplementary concept word, rare disease supplementary concept word, unique identifier, synonyms, population supplementary concept word, anatomy supplementary concept word] 48165

17 (unstable adj3 angina).mp. [mp=title, book title, abstract, original title, name of substance word, subject heading word, floating sub-heading word, keyword heading word, organism supplementary concept word, protocol supplementary concept word, rare disease supplementary concept word, unique identifier, synonyms, population supplementary concept word, anatomy supplementary concept word] 18273

18 unstable coronary.mp. [mp=title, book title, abstract, original title, name of substance word, subject heading word, floating sub-heading word, keyword heading word, organism supplementary concept word, protocol supplementary concept word, rare disease supplementary concept word, unique identifier, synonyms, population supplementary concept word, anatomy supplementary concept word] 885

19 1 or 2 or 3 or 4 or 5 or 6 or 7 or 8 or 9 or 10 or 11 or 12 or 13 or 14 or 15 or 16 or 17 or 18 351706

20 cerebrovascular disorders/ or basal ganglia cerebrovascular disease/ or brain ischemia/ 109973

21 exp Brain Infarction/ 43500

22 Hypoxia-Ischemia, Brain/ 7195

23 carotid artery diseases/ or carotid artery thrombosis/ 26338

24 Carotid Artery, Internal, Dissection/ 1462

25 Intracranial Arterial Diseases/ 406

26 Cerebral Arterial Diseases/ 2720

27 cerebral infarction/ or infarction, anterior cerebral artery/ or infarction, middle cerebral artery/ or infarction, posterior cerebral artery/ 35184

28 exp "Intracranial Embolism and Thrombosis"/ 22705

29 exp Stroke/ 179320

30 Vertebral Artery Dissection/ 1520

31 ((brain or cerebr$ or cerebell$ or vertebrobasil$ or hemispher$ or intracran$ or intracerebral or infratentorial or supratentorial or middle cerebr$ or mca$ or anterior circulation or basilar artery or vertebral artery) adj5 (isch?emi$ or infarct$ or thrombo$ or emboli$ or occlus$)).mp. [mp=title, book title, abstract, original title, name of substance word, subject heading word, floating sub-heading word, keyword heading word, organism supplementary concept word, protocol supplementary concept word, rare disease supplementary concept word, unique identifier, synonyms, population supplementary concept word, anatomy supplementary concept word] 193234

32 20 or 21 or 22 or 23 or 24 or 25 or 26 or 27 or 28 or 29 or 30 or 31 352463

33 exp Heart Failure/ 152036

34 Cardiomyopathy, Dilated/ 17404

35 Shock, Cardiogenic/ 11108

36 exp Ventricular Dysfunction/ 44049

37 Cardiac Output, Low/ 5622

38 ((heart or cardiac or myocardial) adj2 (failure or decompensation)).mp. [mp=title, book title, abstract, original title, name of substance word, subject heading word, floating sub-heading word, keyword heading word, organism supplementary concept word, protocol supplementary concept word, rare disease supplementary concept word, unique identifier, synonyms, population supplementary concept word, anatomy supplementary concept word] 275203

39 ((congestive or acute or decompensat* or chronic) adj2 heart failure).mp. [mp=title, book title, abstract, original title, name of substance word, subject heading word, floating sub-heading word, keyword heading word, organism supplementary concept word, protocol supplementary concept word, rare disease supplementary concept word, unique identifier, synonyms, population supplementary concept word, anatomy supplementary concept word] 79310

40 ((dilated or congestive) adj2 cardiomyopath*).mp. [mp=title, book title, abstract, original title, name of substance word, subject heading word, floating sub-heading word, keyword heading word, organism supplementary concept word, protocol supplementary concept word, rare disease supplementary concept word, unique identifier, synonyms, population supplementary concept word, anatomy supplementary concept word] 27211

41 cardiogenic shock.mp. [mp=title, book title, abstract, original title, name of substance word, subject heading word, floating sub-heading word, keyword heading word, organism supplementary concept word, protocol supplementary concept word, rare disease supplementary concept word, unique identifier, synonyms, population supplementary concept word, anatomy supplementary concept word] 16000

42 ((ventricular or ventricle*) adj2 (failure or insufficien* or dysfunction*)).mp. [mp=title, book title, abstract, original title, name of substance word, subject heading word, floating sub-heading word, keyword heading word, organism supplementary concept word, protocol supplementary concept word, rare disease supplementary concept word, unique identifier, synonyms, population supplementary concept word, anatomy supplementary concept word] 63481

43 lvsd.mp. [mp=title, book title, abstract, original title, name of substance word, subject heading word, floating sub-heading word, keyword heading word, organism supplementary concept word, protocol supplementary concept word, rare disease supplementary concept word, unique identifier, synonyms, population supplementary concept word, anatomy supplementary concept word] 696

44 (acute adj2 heart failure).mp. [mp=title, book title, abstract, original title, name of substance word, subject heading word, floating sub-heading word, keyword heading word, organism supplementary concept word, protocol supplementary concept word, rare disease supplementary concept word, unique identifier, synonyms, population supplementary concept word, anatomy supplementary concept word] 12941

45 33 or 34 or 35 or 36 or 37 or 38 or 39 or 40 or 41 or 42 or 43 or 44 348374

46 exp Hyperglycemia/ 41974

47 hyperglyc?emi*.mp. [mp=title, book title, abstract, original title, name of substance word, subject heading word, floating sub-heading word, keyword heading word, organism supplementary concept word, protocol supplementary concept word, rare disease supplementary concept word, unique identifier, synonyms, population supplementary concept word, anatomy supplementary concept word] 87903

48 glyc?emi*.mp. [mp=title, book title, abstract, original title, name of substance word, subject heading word, floating sub-heading word, keyword heading word, organism supplementary concept word, protocol supplementary concept word, rare disease supplementary concept word, unique identifier, synonyms, population supplementary concept word, anatomy supplementary concept word] 77420

49 elevated glucose.mp. [mp=title, book title, abstract, original title, name of substance word, subject heading word, floating sub-heading word, keyword heading word, organism supplementary concept word, protocol supplementary concept word, rare disease supplementary concept word, unique identifier, synonyms, population supplementary concept word, anatomy supplementary concept word] 2184

50 46 or 47 or 48 or 49 160180

51 relative.mp. [mp=title, book title, abstract, original title, name of substance word, subject heading word, floating sub-heading word, keyword heading word, organism supplementary concept word, protocol supplementary concept word, rare disease supplementary concept word, unique identifier, synonyms, population supplementary concept word, anatomy supplementary concept word] 1038077

52 stress.mp. [mp=title, book title, abstract, original title, name of substance word, subject heading word, floating sub-heading word, keyword heading word, organism supplementary concept word, protocol supplementary concept word, rare disease supplementary concept word, unique identifier, synonyms, population supplementary concept word, anatomy supplementary concept word] 1207029

53 ratio.mp. [mp=title, book title, abstract, original title, name of substance word, subject heading word, floating sub-heading word, keyword heading word, organism supplementary concept word, protocol supplementary concept word, rare disease supplementary concept word, unique identifier, synonyms, population supplementary concept word, anatomy supplementary concept word] 1470893

54 50 and 51 6616

55 52 and 53 55934

56 50 and 55 1051

57 stress hyperglyc?emi* ratio.mp. [mp=title, book title, abstract, original title, name of substance word, subject heading word, floating sub-heading word, keyword heading word, organism supplementary concept word, protocol supplementary concept word, rare disease supplementary concept word, unique identifier, synonyms, population supplementary concept word, anatomy supplementary concept word] 118

58 54 or 56 or 57 7595

59 19 or 32 or 45 963288

60 58 and 59 559

61 limit 60 to humans 453

**Web of science search**

*Timespan: 1970-01-01 to 2024-03-01 (Publication Date)*

**'Myocardial Infarction' OR 'Coronary thrombosis' OR 'acute coronary syndrome' OR 'cerebrovascular disease' OR 'stroke' OR 'brain infarction' OR 'brain ischa*mia' OR 'heart failure' OR 'cardiac failure' OR 'cardiogenic shock'** (Topic)

AND

**stress hyperglyc*emi* ratio' OR 'relative hyperglyc*emia'** (Topic)

782 records

**COCHRANE CENTRAL search**

ID    Search

#1    MeSH descriptor: [Myocardial Infarction] explode all trees

#2    MeSH descriptor: [Coronary Thrombosis] explode all trees

#3    "acute coronary"

#4    MeSH descriptor: [Angina, Unstable] explode all trees

#5    ("heart infarct") (Word variations have been searched)

#6    ("cardiac infarct") (Word variations have been searched)

#7    ("heart attack") (Word variations have been searched)

#8    ("heart muscle infarction") (Word variations have been searched)

#9    ("myocardium infarct*") (Word variations have been searched)

#10   ("subendocardial infarct*") (Word variations have been searched)

#11   ("transmural cardiac infarct*") (Word variations have been searched)

#12   ("transmural heart infarct*") (Word variations have been searched)

#13   ("acs") (Word variations have been searched)

#14   ("ami") (Word variations have been searched)

#15   (coronary near/3 syndrome) (Word variations have been searched)

#16   (unstable near/3 angina) (Word variations have been searched)

#17   (unstable coronary) (Word variations have been searched)

#18   #1 OR #2 OR #3 OR #4 OR #5 OR #6 OR #7 OR #8 OR #9 OR #10 OR #11 OR #12 OR #13 OR #14 OR #15 OR #16 OR #17

#19   MeSH descriptor: [Brain Infarction] explode all trees

#20   MeSH descriptor: [Ischemic Stroke] explode all trees

#21   MeSH descriptor: [undefined] explode all trees

#22   MeSH descriptor: [Infarction, Middle Cerebral Artery] this term only

#23   MeSH descriptor: [Infarction, Posterior Cerebral Artery] this term only

#24   (((brain or cerebr* or cerebell* or vertebrobasil* or hemispher* or intracran* or intracerebral or infratentorial or supratentorial or middle cerebr* or mca* or anterior circulation or basilar artery of vertebral artery) near/5 (isch*emi* or infarct* or thrombo* or emboli* or occlus*))) (Word variations have been searched)

#25   ("CVA") (Word variations have been searched)

#26   ((isch*mi* near/5 (stroke or cerebral vasc* or cerebrovasc* or cva))) (Word variations have been searched)

#27   #19 OR #20 OR #21 OR #22 OR #23 OR #24 OR #25 OR #26

#28   MeSH descriptor: [Heart Failure] explode all trees

#29   MeSH descriptor: [Cardiomyopathy, Dilated] explode all trees

#30   MeSH descriptor: [Shock, Cardiogenic] explode all trees

#31   MeSH descriptor: [Ventricular Dysfunction] explode all trees

#32   MeSH descriptor: [Cardiac Output, Low] explode all trees

#33   (((heart or cardiac or myocardial) next (failure or decompensation))) (Word variations have been searched)

#34   (((congestive or acute) next ("heart failure"))) (Word variations have been searched)

#35   ("cardiogenic shock") (Word variations have been searched)

#36   (((ventricular or ventricle) next (failure or insufficienc* or dysfunction*))) (Word variations have been searched)

#37   ("LVSD") (Word variations have been searched)

#38   #28 OR #29 OR #30 OR #31 OR #32 OR #32 OR #33 OR #34 OR #35 OR #36 OR #37

#39   MeSH descriptor: [Hyperglycemia] explode all trees

#40   ("stress hyperglyc*mi* ratio") (Word variations have been searched)

#41   (stress) (Word variations have been searched)

#42   (ratio) (Word variations have been searched)

#43   (relative) (Word variations have been searched)

#44   (hyperglyc*mi*) (Word variations have been searched)

#45   (glyc*mi*) (Word variations have been searched)

#46   #39 OR #44 OR #45

#47   #41 AND #42 AND #46

#48   #43 AND #39

#49   #47 OR #48 OR #40

#50   #18 OR #27 OR #38

#51   #50 AND #49

**Table 1: List of studies excluded at the full text screening stage**

| **Studies calculated SHR using fasting glucose** |
| --- |
| Merlino G, Pez S, Tereshko Y, Gigli GL, Lorenzut S, Surcinelli A, Valente M. Stress Hyperglycemia Does Not Affect Clinical Outcome of Diabetic Patients Receiving Intravenous Thrombolysis for Acute Ischemic Stroke. Front Neurol. 2022 Jun 13;13:903987. |
| Wang Z, Fan L. Does stress hyperglycemia in diabetic and non-diabetic acute ischemic stroke patients predict unfavorable outcomes following endovascular treatment? Neurol Sci. 2023 May;44(5):1695-1702. doi: 10.1007/s10072-023-06625-y. Epub 2023 Jan 18. |
| Dai Z, Cao H, Wang F, Li L, Guo H, Zhang X, Jiang H, Zhu J, Jiang Y, Liu D, Xu G. Impacts of stress hyperglycemia ratio on early neurological deterioration and functional outcome after endovascular treatment in patients with acute ischemic stroke. Front Endocrinol (Lausanne). 2023 Jan 26;14:1094353. |
| Deng Y, Wu S, Liu J, Liu M, Wang L, Wan J, Zhang S, Liu M. The stress hyperglycemia ratio is associated with the development of cerebral edema and poor functional outcome in patients with acute cerebral infarction. Front Aging Neurosci. 2022 Sep 1;14:936862. |
| Liu C, Zhu XP, Zhu XW, Jiang YM, Xi GJ, Xu L. The acute-to-chronic glycemic ratio correlates with the severity of illness at admission in patients with diabetes experiencing acute ischemic stroke. Front Neurol. 2022 Nov 7;13:938612. |
| Merlino G, Smeralda C, Gigli GL, Lorenzut S, Pez S, Surcinelli A, Marini A, Valente M. Stress hyperglycemia is predictive of worse outcome in patients with acute ischemic stroke undergoing intravenous thrombolysis. J Thromb Thrombolysis. 2021 Apr;51(3):789-797.. |
| Shao T, Liu H, Yang G, Wang H, Li D, Ni H, Xu Y, Zhang J. Fasting blood glucose-to-glycated hemoglobin ratio for evaluating clinical outcomes in patients with ischemic stroke. Front Neurol. 2023 Mar 20;14:1142084. |
| Merlino G, Pez S, Gigli GL, Sponza M, Lorenzut S, Surcinelli A, Smeralda C, Valente M. Stress Hyperglycemia in Patients With Acute Ischemic Stroke Due to Large Vessel Occlusion Undergoing Mechanical Thrombectomy. Front Neurol. 2021 Sep 29;12:725002. |
| Sun, Y., Guo, Y., Ji, Y. *et al.* New stress-induced hyperglycaemia markers predict prognosis in patients after mechanical thrombectomy. *BMC Neurol* **23**, 132 (2023). https://doi.org/10.1186/s12883-023-03175-w |
| Chen X, Liu Z, Miao J, Zheng W, Yang Q, Ye X, Zhuang X, Peng F. High Stress Hyperglycemia Ratio Predicts Poor Outcome after Mechanical Thrombectomy for Ischemic Stroke. J Stroke Cerebrovasc Dis. 2019 Jun;28(6):1668-1673. doi: 10.1016/j.jstrokecerebrovasdis.2019.02.022. Epub 2019 Mar 16. |
| Gu M, Fan J, Xu P, Xiao L, Wang J, Li M, Liu C, Luo G, Cai Q, Liu D, Ye L, Zhou J, Sun W. Effects of perioperative glycemic indicators on outcomes of endovascular treatment for vertebrobasilar artery occlusion. Front Endocrinol (Lausanne). 2022 Oct 5;13:1000030. |
| J N Ngiam, C W S Cheong, A S T Leow, Y -T Wei, J K X Thet, I Y S Lee, C -H Sia, B Y Q Tan, C -M Khoo, V K Sharma, L L L Yeo, Stress hyperglycaemia is associated with poor functional outcomes in patients with acute ischaemic stroke after intravenous thrombolysis, QJM: An International Journal of Medicine, Volume 115, Issue 1, January 2022, Pages 7–11, <https://doi.org/10.1093/qjmed/hcaa253> |
| Zhu B, Pan Y, Jing J, Meng X, Zhao X, Liu L, Wang Y, Wang Y, Wang Z. Stress Hyperglycemia and Outcome of Non-diabetic Patients After Acute Ischemic Stroke. Front Neurol. 2019 Sep 18;10:1003. |
| Wang L, Cheng Q, Hu T, Wang N, Wei X, Wu T, Bi X. Impact of Stress Hyperglycemia on Early Neurological Deterioration in Acute Ischemic Stroke Patients Treated With Intravenous Thrombolysis. Front Neurol. 2022 May 13;13:870872. |
| Luo J, Xu S, Li H, Li Z, Gong M, Qin X, Zhang X, Hao C, Liu X, Zhang W, Xu W, Liu B, Wei Y. Prognostic impact of stress hyperglycemia ratio in acute myocardial infarction patients with and without diabetes mellitus. Nutr Metab Cardiovasc Dis. 2022 Oct;32(10):2356-2366. |
| Cai, Zm., Zhang, Mm., Feng, Rq. *et al.* Fasting blood glucose-to-glycated hemoglobin ratio and all-cause mortality among Chinese in-hospital patients with acute stroke: a 12-month follow-up study. *BMC Geriatr* **22**, 508 (2022). |
| Zhang J, Dong D, Zeng Y, Yang B, Li F, Chen X, Lu J, Guan M, He N, Qiao H, Li K, Xu A, Huang L, Zhu H. The association between stress hyperglycemia and unfavorable outcomes in patients with anterior circulation stroke after mechanical thrombectomy. Front Aging Neurosci. 2023 Jan 5;14:1071377. |
| Fu R, Cui K, Yang J, Xu H, Yin D, Song W, Wang H, Zhu C, Feng L, Wang Z, Wang Q, Lu Y, Dou K, Yang Y; CAMI Registry Investigators. Fasting stress hyperglycemia ratio and in-hospital mortality after acute myocardial infarction in patients with different glucose metabolism status: Results from China acute myocardial infarction registry. Diabetes Res Clin Pract. 2023 Feb;196:110241. Epub 2023 Jan 6. |
| Li J, Quan K, Wang Y, Zhao X, Li Z, Pan Y, Li H, Liu L, Wang Y. Effect of Stress Hyperglycemia on Neurological Deficit and Mortality in the Acute Ischemic Stroke People With and Without Diabetes. Front Neurol. 2020 Sep 24;11:576895. |
| Mi D, Li Z, Gu H, Jiang Y, Zhao X, Wang Y, Wang Y. Stress hyperglycemia is associated with in-hospital mortality in patients with diabetes and acute ischemic stroke. CNS Neurosci Ther. 2022 Mar;28(3):372-381. |
| Liu X, Nie XM, Pu YH, Yan HY, Pan YS, Liu LP. [The association between stress hyperglycemia ratio and outcome of patients with acute ischemic stroke undergoing endovascular treatment]. Zhonghua Yi Xue Za Zhi. 2022 Jul 19;102(27):2096-2102. Chinese. |
| **Alternative study outcomes** |
| Cannarsa GJ, Wessell AP, Chryssikos T, Stokum JA, Kim K, De Paula Carvalho H, Miller TR, Morris N, Badjatia N, Chaturvedi S, Gandhi D, Simard JM, Jindal G. Initial Stress Hyperglycemia Is Associated With Malignant Cerebral Edema, Hemorrhage, and Poor Functional Outcome After Mechanical Thrombectomy. Neurosurgery. 2022 Jan 1;90(1):66-71. |
| Şimşek B, Çınar T, Tanık VO, İnan D, Zeren G, Avcı İİ, Güngör B, Yılmaz F, Tanboğa İH, Karabay CY. The association of acute--to--chronic glycemic ratio with no-reflow in patients with ST--segment elevation myocardial infarction undergoing primary percutaneous coronary intervention. Kardiol Pol. 2021 Feb 25;79(2):170-178. |
| Roberts GW, Larwood C, Krinsley JS. Quantification of stress-induced hyperglycaemia associated with key diagnostic categories using the stress hyperglycaemia ratio. Diabet Med. 2022 Oct;39(10):e14930. Epub 2022 Aug 9. |
| Li G, Wang C, Wang S, Hao Y, Xiong Y, Zhao X. Clinical Significance of Stress Hyperglycemic Ratio and Glycemic Gap in Ischemic Stroke Patients Treated with Intravenous Thrombolysis. *Clin Interv Aging*. 2022;17:1841-1849 |
| Qi S, Jin Z, Zhu Y, Li X.THE STRESS HYPERGLYCEMIA RATIO IS A MARKER FOR AN INCREASED RISK OF MYOCARDIAL ISCHEMIA-REPERFUSION INJURY AFTER MYOCARDIAL INFARCTION. Acta Medica Mediterranea 2023;39(2):565-572 |
| **Conference abstract** |
| Wang Y, Li J, Li Y, Liu T, Fan H, Zhang K, Niu X. ASSOCIATION OF STRESS HYPERGLYCEMIA AND ICAS WITH OUTCOMES OF MINOR STROKE DURING HOSPITALIZATION. International Journal of Stroke 2022;17(3 Supplement):40-42 |
| Takayuki K, Shungo H, Daisaku N, Hiroya M, Shinichiro S, Katsuki O, Tetsuhisa K, Tomoharu D, Hirota K, Bolrathanak O, Akihiro S, Yasushi S. The impact of stress hyperglycemia ratio on long-term outcomes in St segment elevation myocardial infarction patients without diabetes mellitus. Circulation 2018;138(Supplement 1) |
| Campodonico J, Cosentino N, Milazzo V, De Metrio M, Rubino M, Moltrasio M, Marana I, Grazi M, Lauri G, Marenzi G. Prognostic value of the acute to chronic glycemic ratio at admission in acute myocardial infarction. Circulation 2017;136(Supplement 1): |
| Zeng, G, Yuan, D, Yuan, J. TCTAP A-022 Association of Stress Hyperglycemia With Long-Term Mortality After Acute Myocardial Infarction in Patients With or Without Diabetes.. *J Am Coll Cardiol.*2023 Apr, 81 (16_Supplement) |
| I Teraguchi, T Imanishi, K Komukai, T Tamaki, S Imamura, H Kataiwa, The association between acute-to-chronic glycemic ratio and outcome in acute heart failure, European Heart Journal, Volume 41, Issue Supplement_2, November 2020, ehaa946.1206, |
| **Conference presentation** |
| Cheong C, Yuen T.W, Thet J.K, Lee I.Y, Tan B.Y, Sia C.H, Sharma V.K, Yeo L.L.Stress Hyperglycaemia Associated with Poor Functional Outcomes in Acute Ischaemic Stroke Patients treated with Intravenous Thrombolysis. European Journal of Neurology 2020;27(Supplement 1):119 |
| Mazza M, Moro M, Puglia D, De Metrio M, Marenzi G, Acquaviva S, Gusmaroli A, Mattavelli E, Sponton A.  Acute to chronic glycemic ratio at hospital admission in AMI patients and diabetes mellitus: A new indicator of assistance complexity. European Journal of Cardiovascular Nursing 2018;17(1 Supplement 1):86 |
| **Correspondence** |
| Gao S, Liu Q, Ding X, Chen H, Zhao X, Li H. Predictive Value of the Acute-to-Chronic Glycemic Ratio for In-Hospital Outcomes in Patients With ST-Segment Elevation Myocardial Infarction Undergoing Percutaneous Coronary Intervention. *Angiology*. 2020;71(1):38-47. |
| Sun C, Qin F, Zhao Y, Bai F, Liu N, Liu Z, Liu Q. Could the stress hyperglycemia ratio predict the clinical outcomes of coronary artery disease patients after percutaneous coronary intervention? Int J Cardiol. 2018 Mar 1;254:343. Epub 2018 Jan 28. |
| **Poster** |
| J Alves Guimaraes, F M Goncalves, S Borges, J J Monteiro, P S Mateus, J Trigo, J I Moreira, P6442 The acute on chronic glycaemic ratio: a simple tool to stratify risk in acute coronary syndrome patients, European Heart Journal, Volume 40, Issue Supplement_1, October 2019 |
| N. Cosentino, V. Milazzo, M. De Metrio, M. Cecere, S. Mosca, M. Rubino, J. Campodonico, I. Marana, M. Grazi, M. Moltrasio, G. Lauri, G. Marenzi, P2770 In-hospital prognostic relevance of acute on chronic glycemic ratio in patients with acute myocardial infarction, European Heart Journal, Volume 38, Issue suppl_1, August 2017, |
| **Calculated SHR with average 24-hour admission glucose** |
| Lee TF, Burt MG, Heilbronn LK, Mangoni AA, Wong VW, McLean M, Cheung NW. Relative hyperglycemia is associated with complications following an acute myocardial infarction: a post-hoc analysis of HI-5 data. Cardiovasc Diabetol. 2017 Dec 12;16(1):157. |
| **No SHR measurement** |
| Garadah TS, Kassab S, Al-Shboul QM, Alawadi A. The threshold of admission glycemia as a predictor of adverse events in diabetic and non-diabetic patients with acute coronary syndrome. Clin Med Cardiol. 2009 Apr 1;3:29-36. |
| **Meta- analysis** |
| Huang YW, Yin XS, Li ZP. Association of the stress hyperglycemia ratio and clinical outcomes in patients with stroke: A systematic review and meta-analysis. Front Neurol. 2022 Sep 1;13:999536. |
| Huang YW, An YH, Yin XS, Li ZP. Association of the stress hyperglycemia ratio and clinical outcomes in patients with cardiovascular diseases: a systematic review and meta-analysis. Eur Rev Med Pharmacol Sci. 2022 Dec;26(24):9258-9269. |
| **No HR/OR provided** |
| Arun K, Santhanam J, Sruthi D, Kumarasamy S, Meenakshi, Sundari S.N. Stress hyperglycemia ratio and its association with outcomes among patients admitted with ST-segment elevation myocardial infarction (STEMI) in Coronary Care Unit: An observational prospective study. Critical Care and Shock 2022;25(5):229-240 |
| Schmitz T, Freuer D, Harmel E, Heier M, Peters A, Linseisen J, Meisinger C. Prognostic value of stress hyperglycemia ratio on short- and long-term mortality after acute myocardial infarction. Acta Diabetol. 2022 Aug;59(8):1019-1029. |
| **Not in English** |
| Zhi-li X, Cheng-jie G, Ya-jie G, Yi-jing T, Qing W, Hao W, Jun-bo W, Yi Z, Jing-wei P . Value of stress hyperglycemia ratio in predicting the prognosis of patients with acute myocardial infarction. Journal of Shanghai Jiaotong University (Medical Science) 2019, 39(3): 309-315 |

**Table 2: Quality assessment (Newcastle-Ottawa quality assessment scale for cohort studies)**

| **Authors** | Representativeness of exposed cohort | Selection of the non-exposed cohort | Ascertainment of exposure | Outcome of interest was not present at start of study | Comparability of the cohorts | Ascertainment of outcome | Follow up long enough for events to occur? | Adequacy of follow up | Total |
| --- | --- | --- | --- | --- | --- | --- | --- | --- | --- |
| Carrera et al, 2021 | * | * | * | * | ** | * | * |  | *********(8) |
| Cunha et al, 2023 | * | * | * | * | * |  | * |  | ********(6) |
| Mohammed et al, 2024 | * | * | * | * | ** | * | * | * | *******(9) |
| Zhou et al, 2023 | * | * | * | * | ** |  | * | * | ********(8) |
| Li et al, 2024 | * | * | * | * | ** |  | * |  | *********(7) |
| Chen et al, 2022 | * | * | * | * | ** | * | * | * | ********(9) |
| Peng et al, 2023 | * | * | * | * | ** | * | * |  | *********(8) |
| Wang et al, 2019 | * | * | * | * | ** | * | * |  | ********(8) |
| Peng et al, 2024 | * | * | * | * | * | * | * | * | ********(8) |
| Cui et al, 2022 | * | * | * | * | ** | * | * |  | *********(8) |
| Kojima et al, 2020 | * | * | * | * | ** | * | * | * | *********(9) |
| Sia et al, 2021 | * | * | * | * | * | * | * |  | ********(7) |
| Xu et al, 2022 | * | * | * | * | ** | * | * |  | *********(8) |
| Xu et al, 2022 | * | * | * | * | ** | * | * |  | *********(8) |
| Zeng et al, 2023 | * | * | * | * | ** | * | * | * | *********(9) |
| Xie et al, 2023 | * | * | * | * | ** | * | * |  | *********(8) |
| Liu et al, 2023 | * | * | * | * | ** | * | * | * | *******(8) |
| Abdu et al, 2023 | * | * | * | * | * | * | * | * | ********(8) |
| Gao et al, 2023 | * | * | * | * | ** | * | * | * | *********(9) |
| Marenzi et al, 2018 | * | * | * | * | * |  | * |  | *******(6) |
| Lin et al, 2023 | * | * | * | * | ** | * | * |  | *********(8) |
| Gao et al, 2019 | * | * | * | * | ** |  | * | * | *********(8) |
| Yang et al, 2022 | * | * | * | * | ** |  | * | * | ********(8) |
| Yang et al, 2017 | * | * | * | * | ** | * | * |  | ********(8) |
| Roberts et al, 2021 |  | * | * | * | * |  | * |  | ******(5) |
| Zhou et al, 2022 | * | * | * | * | ** | * | * |  | *******(8) |

**Table 3: Variables used in adjustment of risk ratios in each study**

| **First author** | Included in meta-analysis (MA) or systematic review (SR) only |  |
| --- | --- | --- |
| Carrera et al, 2021 | MA | Age, sex, hypertension, diabetes, BMI, CKD, Hb, serum albumin BNP, LVEF, stroke, NYHA class, PAD, HF of ischaemic aetiology |
| Cunha et al, 2023 | MA | Estimated chronic blood glucose, age, ischaemic aetiology, GFR, HFpEF, HFmrEF, ACEi or ARB at admission, BB at admission, MRA at admission |
| Mohammed, et al 2024 | MA | Age, sex, smoking, BMI, AF, CKD, diabetes, eGFR, C-reactive protein, NT-proBNP, LVEF, LAVI, e’, E/e’, LVEDD, ACE+ARB, statins, CCB. |
| Zhou et al, 2023 | MA | Age, sex, smoking, BMI, NT-proBNP, TG, LDL-C, Cr, FT3, SBP, LVEF, E/E', coronary heart disease, AF, use of insulin |
| Li et al, 2024 | MA | Age, sex, urine output, HTN, diabetes, AF, acute HF, MI, OMI, stroke, non ischaemic cardiomyopathy, CKD, NT- proBNP, SCr, BUN, HbA1c, history of insulin use, vasopressors, loop diuretics, MV, RRT |
| Zhou et al, 2022 | MA | Age, sex, baseline SBP, baseline eGFR, baseline NT-proBNP, admission department (Department of Cardiology/others), Charlson Comorbidity Index, with or without ischemic heart disease at baseline, use of insulin, use of venous loop diuretics at baseline. |
| Chen et al, 2022 | MA | Age, sex, current smoking, HL, AF, prior stroke, SBP, NIHSS at admission, BMI, diabetes and triglyceride-glucose index. |
| Peng et al, 2023 | MA | Age, sex, smoking, HTN, HL, baseline NIHSS score, occlusion site and stroke aetiology, diabetes and ASPECTS score |
| Wang et al, 2019 | MA | Age, sex, CHD, neutrophil/lymphocyte ratio (>7.07). NIHSS , preop ASPECTS score, puncture to end of revascularisation (>90 mins), sICH, admission glucose ≥11.1mmol/L |
| Peng et al, 2024 | MA | Age, baseline NIHSS score, stroke aetiology, occlusion site, diabetes, IV thrombolysis status, sex, successful recanalisation. |
| Roberts et al, 2021 | MA | CHA2DS2-VASc, r-tPA and glucose level |
| Shen et al, 2021 | SR only | Age, sex, NIHSS, HTN, smoking, previous TIA/stroke, AF, diabetes, HL, SBP, DBP, RBC, platelets |
| Cui et al, 2022 | MA | Age, sex, BMI, STEMI versus NSTEMI, Killip, primary PCI, smoking, HTN, previous MI, PCI or stroke, CKD, HR, SBP, LVEF, TG, LDL-C, statin, insulin use |
| Kojima et al, 2020 | MA | Age, male sex, BMI, emergency PCI, HTN, dyslipidaemia,, old MI, peak CK on admission, BB/ACEi/ARB at discharge |
| Sia et al, 2021 | MA | Age, history of IHD, Killip class on admission, cardiac arrest on admission, creatinine on admission, Hb on admission |
| Xu et al, 2022 | MA | Age, sex, SBP,, HR, Killip class, diabetes, HTN, angina, weight, anterior STE or LBBB, time to treatment >4hours |
| Xu et al, 2022 | MA | Adjusted for age, sex, BMI, SBP, DBP, smoking, drinking, ACS, TC, and eGFR |
| Zeng et al, 2023 | MA | Age, sex, presentation, previous PCI/MI/CABG/stroke, PAD, COPD, smoking, HTN, diabetes, dyslipidaemia, BMI, eGFR, Hb, PCI, syntax score, LVEF |
| Xie et al, 2023 | MA | Age, sex, SBP, DBP, dialysis use, AMI as index presentation, GRACE score, left main disease, 3- vessel disease, moderate or severe calcification, PCI treatment, ACE inhibitor or ARB and CCB. |
| Liu et al, 2023 | MA | Age, sex, HTN, congestive heart failure, CKD, AF, stroke, anaemia, and revascularisation |
| Abdu et al, 2023 | MA | Age, LVEF, AF, CK-MB, NT-proBNP, diabetes |
| Gao et al, 2023 | MA | Age, sex, MI type (NSTEMI or STEMI), HTN, diabetes, dyslipidemia, LVEF and peak TnI |
| Marenzi et al, 2018 | MA | TIMI score |
| Lin et al, 2023 | MA | Age, sex, eGFR, WBC, Anemia, Current smoker, diabetes, HTN, COPD, prior MI, prior PCI, prior stroke, PCI assessment and multi-vessel stenosis |
| Gao et al, 2019 | MA | Age, sex, peak TnI, PCI timing, and Gensini score |
| Yang et al, 2022 | MA | Age, sex, BMI, diagnosis on admission, family history, previous MI, previous PCI, previous CABG, HTN, HL, diabetes, previous stroke, smoking status, LM disease, 3-vessel disease, CTO disease, ISR disease, SYNTAX score, type, number, length, and diameter of stents, IABP application, DBP, LVEF, low T3 syndrome, eGFR, TG, TC, LDL-C, HDL-C, Hb, uric acid, hs-CRP, and oral drugs (DAPT, statin, b-blocker, ACEI/ARB,and CCB) |
| Yang et al, 2017 | MA | Adjusted for the age (≥ 65 yrs), gender, body mass index (≥ 25 kg/m^2^), HTN, diabetes, hypercholesterolemia, current smoking, MDRD-GFR (< 60 mL/min/1.73 m^2^), previous MI, previous PCI, previous CVA, LVEF (< 45%), anemia (male < 13 g/dL, female < 12 g/dL) and multi-vessel disease. |
| Chen et al, 2023 | SR only | GRACE score, sex, uric acid, PCI, diuretics |
| Gao et al, 2021 | SR only | Age, sex, STEMI v NSTEMI, PCI treatment, peak TnI |
| Xiong et al, 2023 | SR only | GRACE score, diuretics |
| Guo et al, 2023 | SR only | Killip score III and IV, arrythmia |
| Wei et al, 2023 | SR only | Ischemia time, age, sex, BMI, HTN, diabetes, HL, smoking status, previous CVD, previous AF, previous stroke, CKD, previous HF, cancer, culprit vessel, multi-vessel disease |

Abbreviations: BMI, body mass index; ACEi, angiotensin-converting-enzyme inhibitor; ACS, acute coronary syndrome; AF, atrial fibrillation; ALBERTA, **Alberta stroke program early CT score; AMI, acute myocardial infarction;** ARB, angiotensin II receptor blocker; BB, beta blocker; BMI, Body mass index; BNP, B-type natriuretic peptide; BUN, blood urea nitrogen; CABG, coronary artery bypass graft; CCB, calcium channel blocker CHD, coronary heart disease; CK, creatine kinase; COPD, chronic obstructive pulmonary disease; Cr, creatinine; CTO, chronic total occlusion: CVA, cerebrovascular accident; CVD, cardiovascular disease; DAPT, dual antiplatelet therapy; DBP, diastolic blood pressure; FT3, free triiodothyronine; eGFR, estimated glomerular filtration rate; GFR, glomerular filtration rate; GRACE, The Global Registry of Acute Coronary Events; Hb, haemoglobin; HbA1c, glycated haemoglobin; HDL-C, high density lipoprotein cholesterol; HF, heart failure; HFmrEF, heart failure with mildly reduced ejection fraction; HFpEF, heart failure with preserved ejection fraction; HL, hyperlipidaemia; HR, heart rate; hs-CRP, high sensitivity C-reative protein; HTN, hypertension; IABP, intra-aortic balloon pump; IHD, ishcaemic heart disease; ISR, in-stent restenosis; LAVI, left atrial volume index; LBBB, left bundle branck block; LDL- C, low density lipoprotein cholesterol; LM, left main; LVEDD, left ventricular end diastolic diameter; LVEF, left ventricular ejection fraction; MACCE, Major Adverse Cardiac and Cerebrovascular Event; MACE, Major Adverse Cardiac Event; MDRD, Modification of Diet in Renal Disease; MI, myocardial infarction; MRA, mineralocorticoid receptor antagonist; MV, mechanical ventilation; NIHSS, National Institutes of Health Stroke Scale; NSTEMI, non-ST elevation myocardial infarction; NT-proBNP, N-terminal pro b-type natriuretic peptide; NYHA, New York Heart Association; OMI, occlusion myocardial infarction; PAD, peripheral artery disease; PCI, percutaneous coronary intervention; RBC, red blood cell; RRT, renal replacement therapy; r-tPA, recombinant tissue plasminogen activator; SBP, systolic blood pressure; SCr, serum creatinine; sICH, symptomatic intracranial hemorrhage; STE, ST segment elevation; STEMI, ST-elevation myocardial infarction;  SYNTAX, SYNergy between percutaneous coronary intervention with TAXus and cardiac surgery; TC, total cholesterol; TIA, transient ischaemic attack; TG, triglyceride; TIMI, Thrombolysis in myocardial infarction; TnI, troponin I; T3, triiodothyronine; WBC, white blood cell.

**Figure 1: Forest plot with HF studies removed**

**Figure 2: Forest plot with low/medium quality studies removed**

**Figure 3: Contour enhanced funnel plot**

**Figure 4: Meta regression for age bubble plot**

**Table 4: Data extraction form**

| Name of study |  |
| --- | --- |
| Location |  |
| Authors |  |
| Type of study |  |
| Recruitment from/ source of data |  |
| Pathology |  |
| Inclusion criteria |  |
| Exclusion criteria |  |
| Number |  |
| Recruitment  Start date  End date |  |
| Mortality outcome time |  |
| Cause of death |  |
| How SHR was calculated |  |
| SHR categories |  |
| How was outcome assessed? |  |
| Age |  |
| Sex |  |
| Risk ratio(s) |  |
| Covariates used for adjustment |  |

**Table 5: PRISMA 2020 Checklist**

| **Section and Topic** | **Item #** | **Checklist item** | **Location where item is reported** |
| --- | --- | --- | --- |
| **TITLE** | | |  |
| Title | 1 | Identify the report as a systematic review. | Page 1 |
| **ABSTRACT** | | |  |
| Abstract | 2 | See the PRISMA 2020 for Abstracts checklist. | Page 2-3 |
| **INTRODUCTION** | | |  |
| Rationale | 3 | Describe the rationale for the review in the context of existing knowledge. | Page 4 |
| Objectives | 4 | Provide an explicit statement of the objective(s) or question(s) the review addresses. | Page 4 |
| **METHODS** | | |  |
| Eligibility criteria | 5 | Specify the inclusion and exclusion criteria for the review and how studies were grouped for the syntheses. | Page 5 |
| Information sources | 6 | Specify all databases, registers, websites, organisations, reference lists and other sources searched or consulted to identify studies. Specify the date when each source was last searched or consulted. | Page 5 |
| Search strategy | 7 | Present the full search strategies for all databases, registers and websites, including any filters and limits used. | Supplement (‘Search strategies’) |
| Selection process | 8 | Specify the methods used to decide whether a study met the inclusion criteria of the review, including how many reviewers screened each record and each report retrieved, whether they worked independently, and if applicable, details of automation tools used in the process. | Pages 5-6 |
| Data collection process | 9 | Specify the methods used to collect data from reports, including how many reviewers collected data from each report, whether they worked independently, any processes for obtaining or confirming data from study investigators, and if applicable, details of automation tools used in the process. | Page 5-6 |
| Data items | 10a | List and define all outcomes for which data were sought. Specify whether all results that were compatible with each outcome domain in each study were sought (e.g. for all measures, time points, analyses), and if not, the methods used to decide which results to collect. | Page 6 |
|  | 10b | List and define all other variables for which data were sought (e.g. participant and intervention characteristics, funding sources). Describe any assumptions made about any missing or unclear information. | Page 6/Table 1 |
| Study risk of bias assessment | 11 | Specify the methods used to assess risk of bias in the included studies, including details of the tool(s) used, how many reviewers assessed each study and whether they worked independently, and if applicable, details of automation tools used in the process. | Page 6 |
| Effect measures | 12 | Specify for each outcome the effect measure(s) (e.g. risk ratio, mean difference) used in the synthesis or presentation of results. | Page 6 |
| Synthesis methods | 13a | Describe the processes used to decide which studies were eligible for each synthesis (e.g. tabulating the study intervention characteristics and comparing against the planned groups for each synthesis (item #5)). | Page 6-7 |
|  | 13b | Describe any methods required to prepare the data for presentation or synthesis, such as handling of missing summary statistics, or data conversions. | - |
|  | 13c | Describe any methods used to tabulate or visually display results of individual studies and syntheses. | Table 4 supplement |
|  | 13d | Describe any methods used to synthesize results and provide a rationale for the choice(s). If meta-analysis was performed, describe the model(s), method(s) to identify the presence and extent of statistical heterogeneity, and software package(s) used. | Pages 6-7 |
|  | 13e | Describe any methods used to explore possible causes of heterogeneity among study results (e.g. subgroup analysis, meta-regression). | Pages 6-7 |
|  | 13f | Describe any sensitivity analyses conducted to assess robustness of the synthesized results. | Pages 6-7 |
| Reporting bias assessment | 14 | Describe any methods used to assess risk of bias due to missing results in a synthesis (arising from reporting biases). | Page 7 |
| Certainty assessment | 15 | Describe any methods used to assess certainty (or confidence) in the body of evidence for an outcome. | - |
| **RESULTS** | | |  |
| Study selection | 16a | Describe the results of the search and selection process, from the number of records identified in the search to the number of studies included in the review, ideally using a flow diagram. | Figure 1 main |
|  | 16b | Cite studies that might appear to meet the inclusion criteria, but which were excluded, and explain why they were excluded. | Figure 1 main, Table 1 supplement |
| Study characteristics | 17 | Cite each included study and present its characteristics. | Table 1 main |
| Risk of bias in studies | 18 | Present assessments of risk of bias for each included study. | Table 2 supplement |
| Results of individual studies | 19 | For all outcomes, present, for each study: (a) summary statistics for each group (where appropriate) and (b) an effect estimate and its precision (e.g. confidence/credible interval), ideally using structured tables or plots. | Figure 2 main |
| Results of syntheses | 20a | For each synthesis, briefly summarise the characteristics and risk of bias among contributing studies. | Page 9-10 |
|  | 20b | Present results of all statistical syntheses conducted. If meta-analysis was done, present for each the summary estimate and its precision (e.g. confidence/credible interval) and measures of statistical heterogeneity. If comparing groups, describe the direction of the effect. | Figure 2,3,4,5 main,, pages 9-10. |
|  | 20c | Present results of all investigations of possible causes of heterogeneity among study results. | Pages 9-10, Figures 3,4,5 main Figures 1 and 2, supplement |
|  | 20d | Present results of all sensitivity analyses conducted to assess the robustness of the synthesized results. | Figure 2, supplement |
| Reporting biases | 21 | Present assessments of risk of bias due to missing results (arising from reporting biases) for each synthesis assessed. | Page 10, Figure 3 supplement |
| Certainty of evidence | 22 | Present assessments of certainty (or confidence) in the body of evidence for each outcome assessed. | - |
| **DISCUSSION** | | |  |
| Discussion | 23a | Provide a general interpretation of the results in the context of other evidence. | Page 14-19 |
|  | 23b | Discuss any limitations of the evidence included in the review. | Page 17-18 |
|  | 23c | Discuss any limitations of the review processes used. | Page 18 |
|  | 23d | Discuss implications of the results for practice, policy, and future research. | Pages 14-19 |
| **OTHER INFORMATION** | | |  |
| Registration and protocol | 24a | Provide registration information for the review, including register name and registration number, or state that the review was not registered. | Page 1 and 5 |
|  | 24b | Indicate where the review protocol can be accessed, or state that a protocol was not prepared. | Page 1 and 5 |
|  | 24c | Describe and explain any amendments to information provided at registration or in the protocol. | - |
| Support | 25 | Describe sources of financial or non-financial support for the review, and the role of the funders or sponsors in the review. | Page 20-21 |
| Competing interests | 26 | Declare any competing interests of review authors. | Page 20 |
| Availability of data, code and other materials | 27 | Report which of the following are publicly available and where they can be found: template data collection forms; data extracted from included studies; data used for all analyses; analytic code; any other materials used in the review. | Page 7, Table 4 supplement |

*From:*  Page MJ, McKenzie JE, Bossuyt PM, Boutron I, Hoffmann TC, Mulrow CD, et al. The PRISMA 2020 statement: an updated guideline for reporting systematic reviews. BMJ 2021;372:n71. doi: 10.1136/bmj.n71
